# Chaperonin-Containing TCP-1 Promotes Cancer Chemoresistance and Metastasis through the AKT-GSK3β-β-Catenin and XIAP-Survivin Pathways

**DOI:** 10.3390/cancers12123865

**Published:** 2020-12-21

**Authors:** Yun-Xun Chang, Yuan-Feng Lin, Chi-Long Chen, Ming-Shyan Huang, Michael Hsiao, Po-Huang Liang

**Affiliations:** 1 Institute of Biochemical Sciences, National Taiwan University, Taipei 10617, Taiwan; d04b46002@ntu.edu.tw; 2 Graduate Institute of Clinical Medicine, College of Medicine, Taipei Medical University, Taipei 11031, Taiwan; d001089012@tmu.edu.tw; 3 Department of Pathology, College of Medicine, Taipei Medical University, Taipei 11031, Taiwan; chencl@tmu.edu.tw; 4 Department of Pathology, Taipei Medical University Hospital, Taipei 11031, Taiwan; 5 Department of Internal Medicine, E-Da Cancer Hospital, School of Medicine, I-Shou University, Kaohsiung 82445, Taiwan; Ed110209@edah.org.tw; 6 Genomics Research Center, Academia Sinica, Taipei 11529, Taiwan; mhsiao@gate.sinica.edu.tw; 7 Institute of Biological Chemistry, Academia Sinica, 128 Academia Road, Taipei 11529, Taiwan

**Keywords:** chaperonin, CCT-β, chemoresistance, metastasis, cancer therapy

## Abstract

**Simple Summary:**

CCT is a chaperonin that participates in folding intracellular proteins. We found that endogenously high expression of the subunit CCT-β is associated with a poorer chemotherapy response in clinical cancer patients. Using two cancer cell lines with higher CCT-β levels, a triple-negative breast cancer cell line MDA-MB-231 and a highly metastatic non-small-cell lung cancer cell line CL1-5, we demonstrated that upregulation of CCT-β expression correlated with chemoresistance and metastasis of these cancer cells in vitro and in vivo. Mechanistic studies allowed us to identify the AKT-GSK3β-β-catenin and XIAP-Survivin pathways promoted by CCT-β to account for the observations. The results provided by our studies are important for developing diagnostic and therapeutic strategies for combating CCT-β-overexpressed cancers.

**Abstract:**

Chaperonin-containing TCP-1 (CCT) is a chaperonin composed of eight subunits that participates in intracellular protein folding. Here, we showed that increased levels of subunits of CCT, particularly CCT-β, were significantly correlated with lower survival rates for cancer patients. Endogenously high expression of CCT-β was found in cancer cell lines, such as the triple-negative breast cancer cell line MDA-MB-231 and the highly metastatic non-small-cell lung cancer cell line CL1-5. Knocking down CCT-β in these cancer cells led to decreased levels of anti-apoptotic proteins, such as XIAP, as well as inhibited phosphorylation of Ser473-AKT and GSK3, resulting in decrease of the nucleus-entering form of β-catenin; these changes reduced the chemoresistance and migration/invasion of the cells. Conversely, overexpression of CCT-β recovered the chemoresistance and cell migration/invasion by promoting the AKT-GSK3β-β-catenin and XIAP-Survivin pathways. Coimmunoprecipitation data revealed that the CCT complex might directly bind and stabilize XIAP and β-catenin. This study not only elucidates the roles of CCT in chemoresistance and metastasis, which are two major obstacles for current cancer therapy, but also provides a possible therapeutic strategy against cancers with overexpressed CCT-β.

## 1. Introduction

Chaperonin-containing TCP-1 (CCT) is an intracellular chaperonin composed of eight subunits (α, β, γ, δ, ε, ζ, η, and θ) that promotes folding of intracellular proteins in cytosol, mainly cytoskeletal proteins, such as tubulin and actin [1,2]. For binding of substrates, CCT forms a barrel-like structure where two back-to-back rings of eight subunits surround a central cavity [3]. In addition to associating with the cytoskeleton, CCT interacts with several proteins in cell cycle regulation [4]. CCT is related to tumorigenesis and proliferation through its role in folding cyclin E, cyclin B, and p21ras [5,6,7,8]. CCT is also involved in cancer metastasis because rearrangement of actin filaments is required for cell migration/invasion, and two actin-regulating proteins, p21-activating kinase PAK4 [9] and gelsolin [10], are bound to CCT. However, CCT seems to inhibit tumor carcinogenesis by binding with the tumor suppressor p53 [11] and the von Hippel-Lindau (VHL) tumor-suppressor protein [12]. Because 10% of intracellular proteins could be substrates of CCT, its roles in cancer and other diseases are not clearly defined.

Among the eight subunits, CCT-β was shown to be related to tumor proliferation and progression [13,14,15], and knockdown of CCT-β appeared to retard the proliferation of tumor cells [16]. A proteomic approach revealed substantially elevated CCT-β expression in chemoresistant cancer cells derived from lung, uterine, and ovarian cancer cell lines compared to their parental cells [17,18]. Enhanced expression of CCT-β in the drug-resistant uterine cancer cell line MES-SA/Dx5 was found; therefore, disrupting the highly expressed CCT-β complex with β-tubulin by a synthetic protein-protein interaction (PPI) inhibitor caused a higher level of apoptosis in MES-SA/Dx5 cells than in MES-SA cells [19]. Later, we showed that the PPI inhibitor also preferentially killed several CCT-β-overexpressing cancer cell lines, including the triple-negative breast cancer (TNBC) cell line MDA-MB-231, the colorectal cancer cell lines Colo205 and HCT116, and the gastric cancer cell line MKN-45 by inducing endoplasmic reticulum (ER) stress, MAPK and caspase activation [20,21]. We further showed that disrupting the interaction of CCT-β with β-tubulin not only induced apoptosis but also inhibited invasion/migration of the highly metastatic non-small-cell lung cancer (NSCLC) cell line CL1-5 [22]. CCT-β upregulation may relive the ER stress caused by the unfolded protein and stabilize its client proteins to suppress apoptotic machinery and potentiate metastatic capability in the cancer cells.

Since CCT-β seems to play a more predominating role than other subunits in cancer chemoresistance and metastasis as shown by previous studies, we used two CCT-β-overexpressing, highly metastatic cancer cell lines, MDA-MB-231 and CL1-5, for knocking down CCT-β and found reduced chemoresistance and cell migration/invasion. Conversely, overexpression of CCT-β in the silenced cells recovered chemoresistance and migration/invasion. Mechanistic studies demonstrated that the AKT-GSK3β-β-catenin and XIAP-Survivin signaling pathways are promoted by overexpressing CCT-β to mediate chemoresistance and metastasis. Since chemoresistance and metastasis are major obstacles for cancer therapy, the results provided by our studies are important for developing diagnostic and therapeutic strategies for combating CCT-β-overexpressed cancers.

## 2. Results

### 2.1. Endogenously High Expression of CCT-β Is Associated with a Poorer Chemotherapy Response in Clinical Cancer Patients

**Figure 1:**
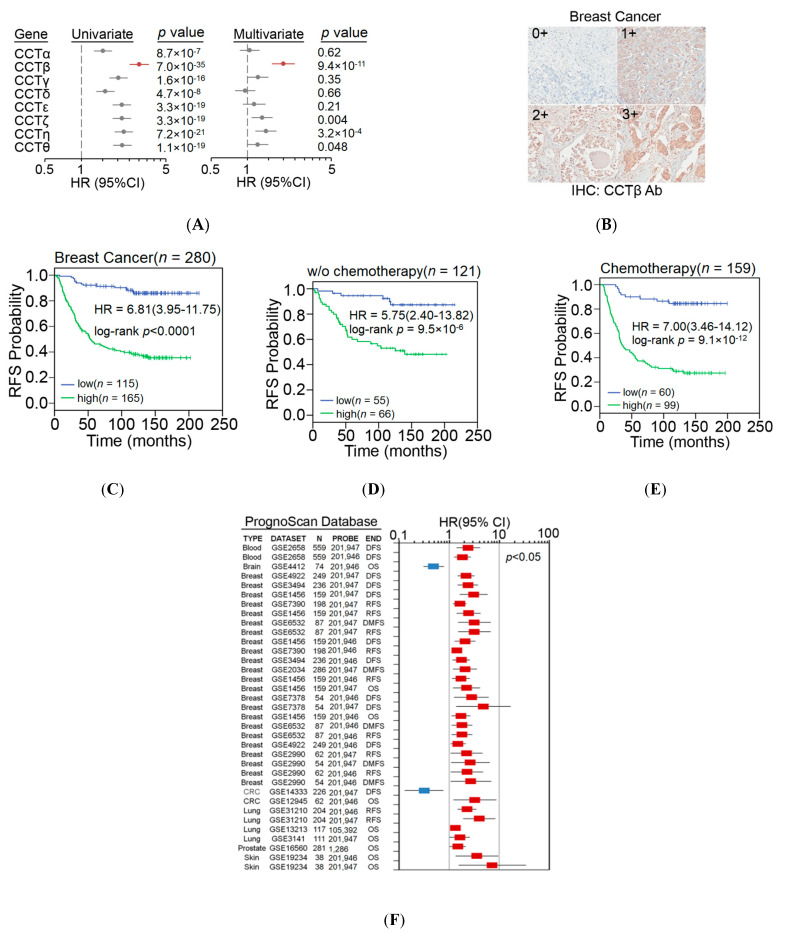
Endogenously high expression of CCT-β indicates poor chemotherapeutic responses in cancer patients. (**A**) Univariate and multivariate analyses under the condition of recurrence-free survival probability against CCT-β gene expression, which were obtained from the Kaplan-Meier Plotter database derived from breast cancer patients who received postoperative chemotherapy. Data are presented as hazard ratios with lower and upper risk values within 95% confidence intervals. (**B**) The intensities of IHC staining for CCT-β protein levels were defined as strong (3+), moderate (2+), weak (1+), or undetectable (0+). (**C**–**E**) Kaplan-Meier analysis of the CCT-β protein levels that were determined by IHC experiments under RFS conditions in a breast cancer cohort that was not classified (**C**) or recorded to be treated without (w/o) (**D**) or with (**E**) systemic postoperative chemotherapy. (**F**) A meta-analysis of the CCT-β gene using the PrognoScan database. Data are presented as hazard ratios with lower and upper risk values within 95% confidence intervals.

We first examined the transcriptional profiling of CCT subunits in Kaplan-Meier Plotter database. Although the upregulation of all CCT components showed a stronger ability to predict poor outcomes in breast cancer patients who received systemic postoperative chemotherapy than in unclassified and untreated patients, multivariate analysis revealed that CCT-β, among all CCT components, was the most useful indicator for predicting a poor chemotherapeutic response (Figure 1A). Here, the univariate analysis of Cox regression test was used to individually evaluate the significance of the detected factor in predicting the risk as shown by hazard ratio (HR) for cancer relapse, while the multivariate analysis was used to estimate the prognostic significance of the detected factor by simultaneously comparing with other listed factors. Moreover, Kaplan-Meyer Plotter data showed that the endogenously high expression of all CCT subunits was associated with poorer recurrence-free survival (RFS) in breast carcinoma, but the effect of CCT-β was greater than that of the others (Figure S1). We then performed survival analysis with a signature derived from the mean expression of all CCT subunits by using RFS, overall survival (OS), distant metastasis-free survival (DMFS), and palliative performance scale (PPS) conditions. The data revealed that a higher level of the signature is significantly correlated with poorer prognoses in these conditions. Particularly in RFS condition, a higher level of the signature refers to the highest HR in the univariate analysis (Figure S2), indicating a critical role of CCT subunits in the mechanism for tumor relapse of breast cancer. As a negative control, survival analysis against PERK, an ER stress-related protein, by using K-M Plotter database for breast cancer (gene chip) was performed to show that PERK expression does not influence RFS condition in the detected breast cancer patients (Figure S3).

We next performed immunohistochemistry (IHC) experiments (Figure 1B) to validate the relevance of endogenously high expression of CCT-β to poor chemosensitivity in clinical cancer patients. Our data showed that endogenously high expression of CCT-β indeed predicted a poor RFS in the breast cancer patients receiving postoperative chemotherapy more powerfully than in the unclassified and untreated patients (Figure 1C–E). Similar results using the K-M Plotter database for analysis are also shown in Figure S4. Notably, endogenously high expression of CCT-β compared to pathologic stage, primary tumor (T) and regional lymph nodes (N), in this breast cancer cohort under the condition of RFS, appeared to be an independent risk factor in a multivariate analysis (Figure S5). Significantly, IHC data revealed that endogenously high expression of CCT-β also served as a poor prognostic marker for patients with lung and colorectal cancer in terms of RFS (Figure S6). Similarly, the increased transcriptional levels of CCT-β appeared to predict unfavorable outcomes in patients with blood, breast, colorectal, lung, prostate, and skin cancer in a global meta-analysis using the PrognoScan database (Figure 1F).

### 2.2. Upregulation of CCT-β Expression Correlated with Chemoresistance in MDA-MB-231 and CL1-5 Cells

**Figure 2:**
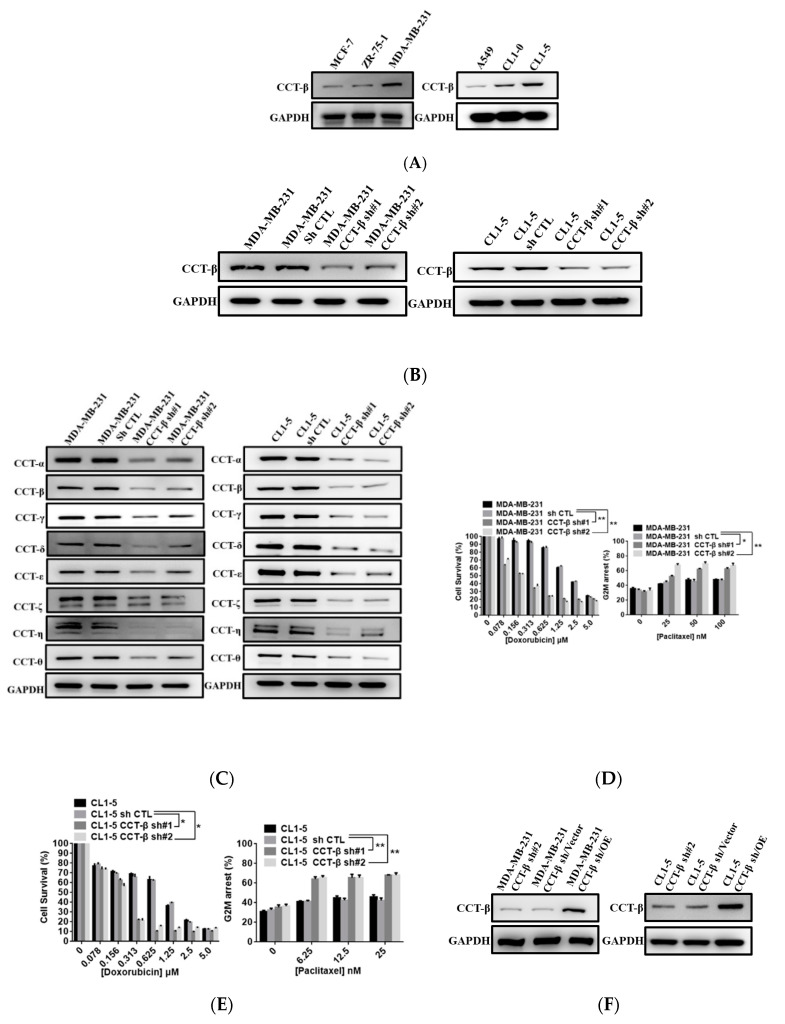

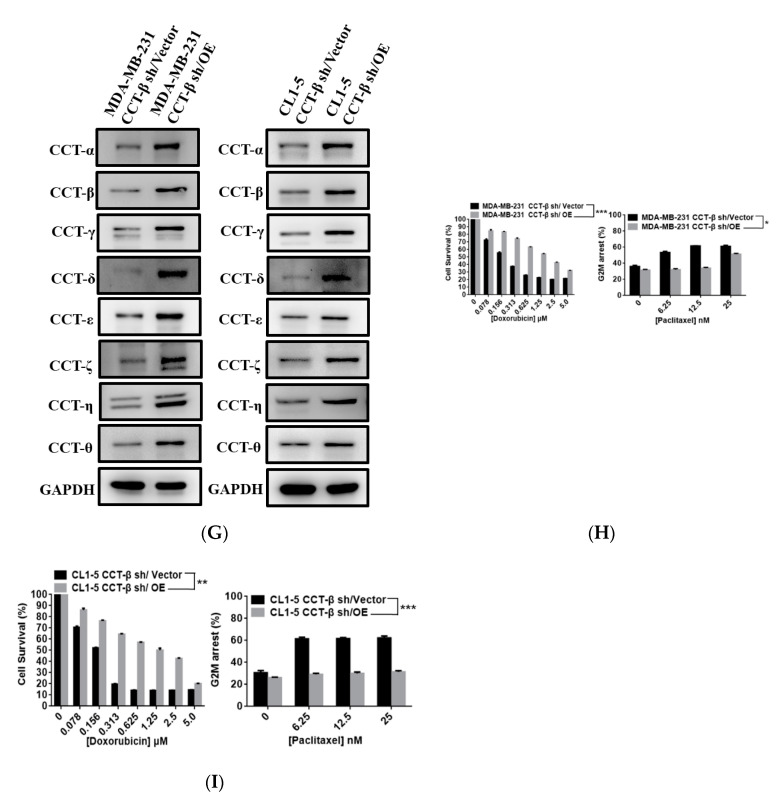
Correlation of drug sensitivity/resistance with CCT-β expression. (**A**) Western blot analysis of the CCT-β expression levels in various breast cancer and lung cancer cell lines. (**B**) The lower expression levels of CCT-β in the MDA-MB-231 and CL1-5 cells after siRNA treatment. (**C**) Western blot analysis of all the CCT subunits in the MDA-MB-231 and CL1-5 cells transfected with the CCT-β siRNAs. (**D**) The apoptotic levels of the MDA-MB-231 cells with and without CCT-β knockdown after doxorubicin and paclitaxel treatments assessed by MTT assays and flow cytometry analysis, respectively. The data showed that the CCT-β-knocked down cells became more sensitive to the anticancer drugs. (**E**) The apoptotic levels of the CL1-5 and CCT-β-silenced cells after doxorubicin and paclitaxel treatments assessed by MTT assays and flow cytometry analysis, respectively. (**F**) The higher expression levels of CCT-β resulted from overexpression of CCT-β in the CCT-β-silenced cells. (**G**) Western blot analysis of all the CCT subunits in the MDA-MB-231 and CL1-5 cells with overexpressed CCT-β from the CCT-β-silenced cells. (**H**) Overexpression of CCT-β in the CCT-β-silenced MDA-MB-231 cells recovered resistance. (**I**) Overexpression of CCT-β in the CCT-β knockdown CL1-5 cells recovered resistance. Data are presented as the mean ± S.D. of three independent experiments. *** *p* < 0.001, ** *p* < 0.01, * *p* < 0.05 vs. the control.

We observed a similar correlation between endogenously high expression of CCT-β and poor chemotherapeutic responses in breast cancers as shown above and lung cancers in previous study [22]. Indeed, higher expression levels of CCT-β were detected in the MDA-MB-231 and CL1-5 cell lines as compared to other breast and lung cancer cell lines, such as MCF-7, ZR-75-1, A549, and CL1-0 (the initial stage and less metastatic form of CL1-5) (Figure 2A). To understand the role of CCT-β in malignancy, we knocked down CCT-β from these two cell lines by using two siRNAs (Figure 2B). CCT-β knockdown in MDA-MB-231 and CL1-5 cells led to a decrease in all other CCT subunits (Figure 2C), indicating a decrease in the CCT complex level. The CCT-β knockdown MDA-MB-231 cells became more sensitive to doxorubicin and paclitaxel (Figure 2D). Similarly, knocking down CCT-β in CL1-5 also increased doxorubicin and paclitaxel sensitivity (Figure 2E). As a control, CCT-β knockdown did not significantly affect the cell survival (Figure S7A,B) and cell cycle according to flow cytometry analysis (Figure S7C,D) in the same time window of 72 h but without a drug treatment. To understand if CCT-β is important in conferring doxorubicin and paclitaxel resistance, we next reconstituted the CCT-β expression in the CCT-β-silenced MB231 and CL1-5 cells by transfecting those cells with exogenous CCT-β gene (Figure 2F). We used shRNA that targets the 3′-untranslated region of CCT-β to generate CCT-β-silencing cells and then transfected these cells with exogenous CCT-β DNA (coding sequence only) lacking the 3′-untranslated region to avoid the hybridization between shRNA and CCT-β transcript in the cells. Overexpression of CCT-β in the CCT-β-silenced MDA-MB-231 and CL1-5 cells recovered the protein levels of all other CCT subunits (Figure 2G). Robustly, the restoration of CCT-β expression in the CCT-β-silenced MDA-MB-MB231 and CL1-5 cells rescued the doxorubicin and paclitaxel resistance (Figure 2H,I).

Our data indicated that the CCT-β expression level correlated with the drug sensitivity/resistance of cancer cells.

### 2.3. Correlation of X-Linked Inhibitor of Apoptosis (XIAP) and β-Catenin Expression with CCT-β Expression

**Figure 3:**
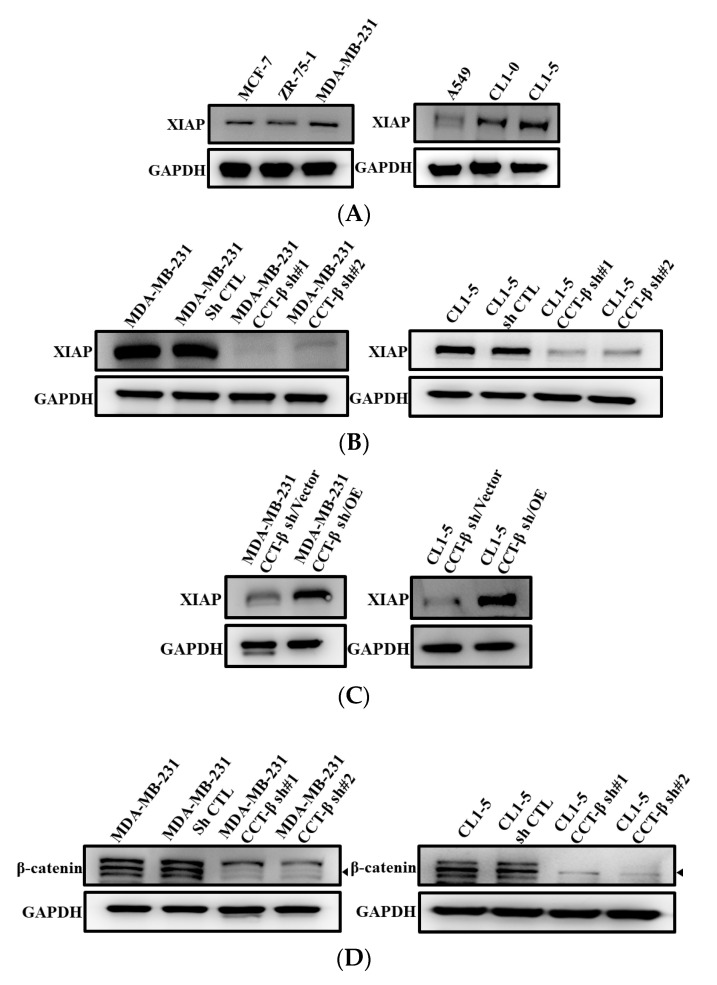

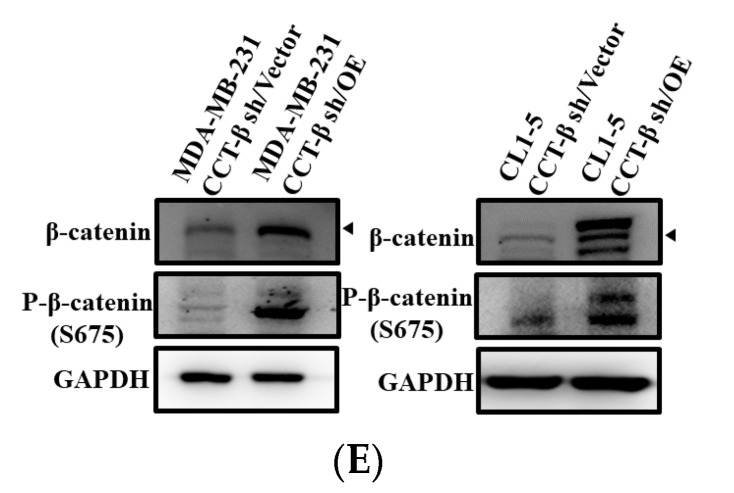
Correlation of XIAP and β-catenin expression with CCT-β expression. (**A**) Expression levels of XIAP in the breast cancer and lung cancer cell lines by western blotting analysis. (**B**) Lower expression levels of XIAP caused by CCT-β knockdown in the MDA-MB-231 and CL1-5 cells. (**C**) Higher expression levels of XIAP induced by overexpression of CCT-β in the CCT-β knockdown cells. (**D**) Lower expression levels of β-catenin caused by CCT-β knockdown in the MDA-MB-231 and CL1-5 cells. (**E**) Higher expression levels of β-catenin and S675-phosphorylated β-catenin were induced by overexpression of CCT-β in the knockdown MDA-MB-231 and CL1-5 cells.

To understand the molecular mechanism of CCT-β overexpression-induced chemoresistance, we examined the expression levels of several key anti-apoptotic proteins. We found that both the MDA-MB-231 and CL1-5 cells had higher levels of XIAP than epithelial breast and lung cells, respectively (Figure 3A). When CCT-β was knocked down, the expression level of XIAP was also reduced (Figure 3B). Overexpression of CCT-β in the CCT-β-silenced cells recovered the XIAP protein level (Figure 3C). Consistently, when CCT-β was knocked down, the expression level of β-catenin was reduced (Figure 3D). Overexpression of CCT-β in the CCT-β-silenced cells recovered the β-catenin and S675-phosphorylated β-catenin protein levels (Figure 3E). Our data clearly indicated that the XIAP and β-catenin expression levels correlated with that of CCT-β. As shown below in detail, CCT complex may directly bind and stabilize XIAP and S675-phosphorylated β-catenin, thereby increasing their levels for causing higher chemoresistance.

### 2.4. Correlation of CCT-β Expression with Chemoresistance in MCF-7 and 7TR

**Figure 4:**
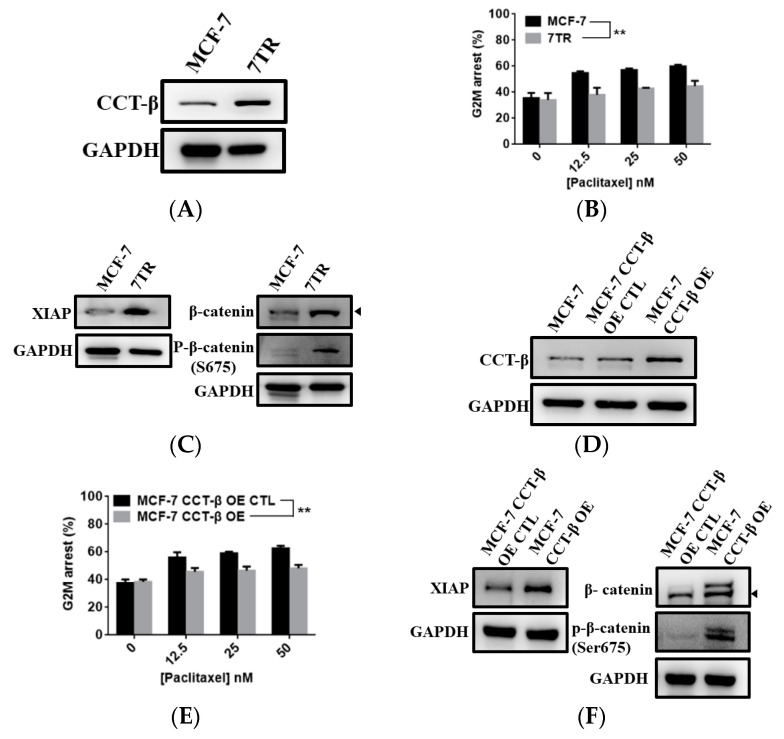

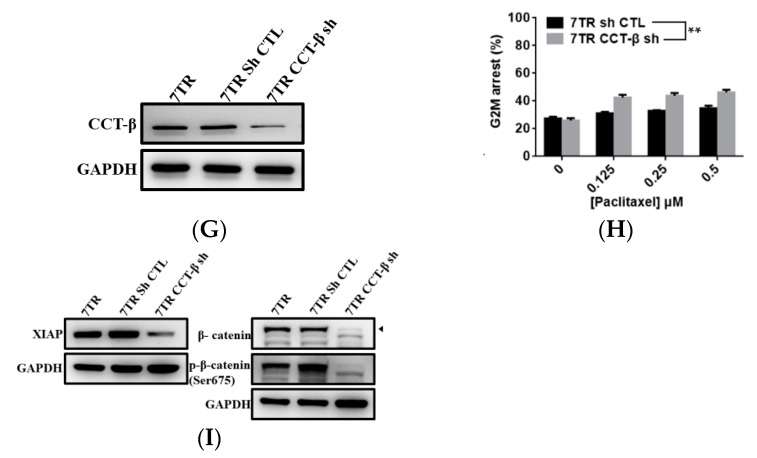
Levels of CCT-β were associated with chemoresistance in MCF-7 and 7TR cells. (**A**) Western blot analysis of the CCT-β expression levels in MCF-7 and 7TR cells. (**B**) The apoptotic levels of MCF-7 and 7TR cells after the paclitaxel treatment assessed by flow cytometry analysis. (**C**) Western blot analysis of the XIAP, β-catenin, and S675-phosphorylated β-catenin expression levels in MCF-7 and 7TR cells. (**D**) Overexpression of CCT-β in MCF-7 cells. (**E**) The apoptotic levels of the MCF-7 cells with and without CCT-β overexpression after the paclitaxel treatment assessed by flow cytometry analysis. (**F**) Higher expression levels of XIAP, β-catenin, and S675-phosphorylated β-catenin caused by CCT-β overexpression in MCF-7 cells. (**G**) Lower expression levels of CCT-β in the siRNA-treated 7TR cells. (**H**) The apoptotic levels of the 7TR cells with and without CCT-β knockdown after the paclitaxel treatment assessed by flow cytometry analysis. (**I**) The lower expression levels of XIAP, β-catenin, and S675-phosphorylated β-catenin caused by CCT-β knockdown in 7TR cells. Data are presented as the mean ± S.D. of three independent experiments. ** *p* < 0.01 vs. the control.

To prove that acquired chemoresistance also requires CCT-β overexpression, we generated a paclitaxel-resistant variant 7TR by exposing MCF-7 breast cancer cells to increasing concentrations of paclitaxel (up to 300 nM). As compared to MCF-7, 7TR cells showed a higher level of CCT-β (Figure 4A) and increased chemoresistance toward paclitaxel (Figure 4B). 7TR cells exhibited higher levels of XIAP, β-catenin, and S675-phosphorylated β-catenin (Figure 4C).

When overexpressing CCT-β from MCF-7 cells (Figure 4D), the cells gained chemoresistance toward paclitaxel (Figure 4E). The higher expression levels of XIAP, β-catenin, and S675-phosphorylated β-catenin were observed in the cells (Figure 4F). Conversely, knocking down CCT-β from 7TR cells (Figure 4G) resulted in reduced chemoresistance (Figure 4H) as well as lower expression levels of XIAP, β-catenin, and S675-phosphorylated β-catenin (Figure 4I).

### 2.5. Knocking down CCT-β Inhibited the Invasion/Migration of MDA-MB-231 and CL1-5 Cells

**Figure 5:**
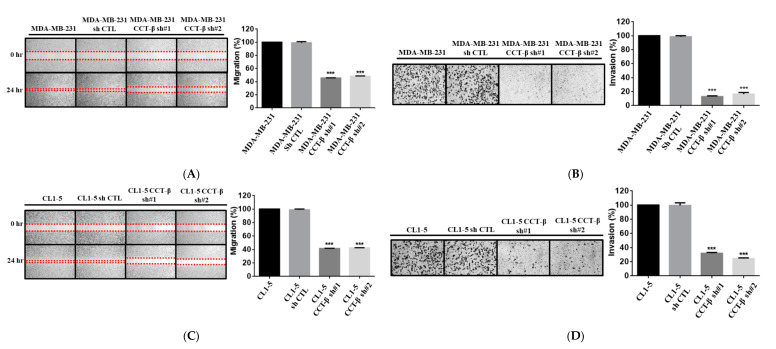

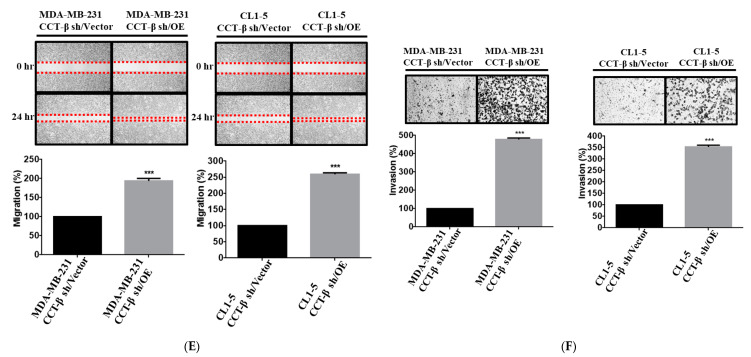
CCT-β knockdown inhibited MDA-MB-231 and CL1-5 cell migration/invasion. (**A**,**C**) Wound-healing assays of the MDA-MB-231 and CL1-5 cells with and without CCT-β silencing. Representative photos for cell migration from the wound edge (the red dotted lines) were captured under a microscope with a 4× objective, and the migratory areas were measured and analyzed using ImageJ software. Data are presented as the mean ± S.D. of three independent experiments. *** *p* < 0.001 vs. the control. (**B**,**D**) Transwell assays for MDA-MB-231 and CL1-5 cells with and without CCT-β silencing. Representative photos of the invasion were captured under a microscope with a 10× objective, and the areas of invaded cells were calculated. Data are presented as the mean ± S.D. of three independent experiments. *** *p* < 0.001 vs. the control. (**E**) Increased wound healing by overexpressing CCT-β in the CCT-β knockdown MDA-MB-231 and CL1-5 cells. (**F**) Transwell assays after overexpression of CCT-β in the CCT-β knockdown MDA-MB-231 and CL1-5 cells.

XIAP and β-catenin are related to cell migration and invasion. To analyze whether MDA-MB-231 and CL1-5 cell metastasis could be regulated by CCT-β through XIAP and β-catenin, we knocked down CCT-β from these two highly metastatic cancer cells. In the MDA-MB-231 cell line, the siRNA-treated cells had decreased levels of cell migration (Figure 5A) and invasion (Figure 5B). Similarly, knocking down CCT-β in CL1-5 cells also reduced cell migration (Figure 5C) and invasion (Figure 5D). Then, overexpression of CCT-β in the CCT-β knockdown MDA-MB-231 and CL1-5 cells recovered cell migration (Figure 5E) and invasion (Figure 5F).

### 2.6. Knocking down CCT-β Inhibited MDA-MB-231 Cell Metastasis In Vivo

**Figure 6:**
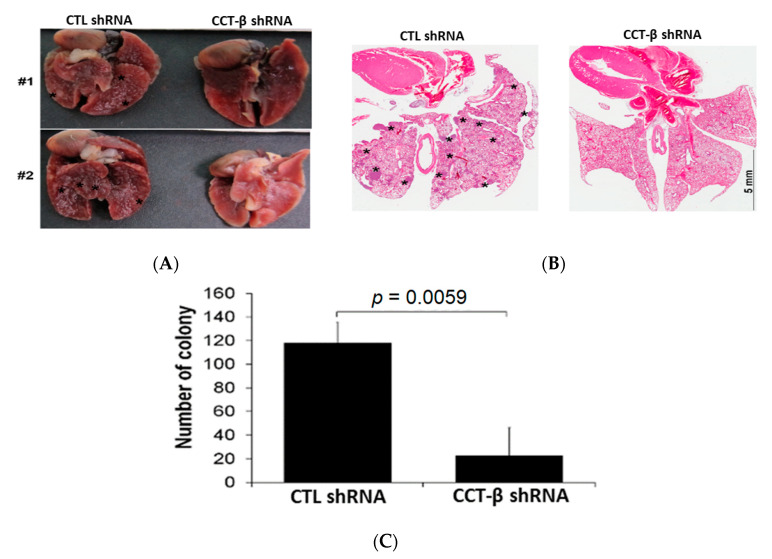
CCT-β knockdown suppressed lung colony formation through the migration of MDA-MB-231 cells in tumor-bearing mice. (**A**) The appearance of tumor nodules in the lungs derived from 2 representative mice transplanted with MDA-MB-231 cells without or with CCT-β knockdown. (**B**) H/E staining for tumor colonies in the lungs from mice transplanted with MDA-MB-231 cells without or with CCT-β knockdown. (**C**) The colony number from the tumor-bearing mice (*n* = 5) is presented as the mean ± SEM. The Mann-Whitney U test was used to analyze the statistical significance.

To validate the role of CCT-β in cancer metastasis, we injected MDA-MB-231 cells with or without CCT-β knockdown into nude mice through the tail vein. As shown in Figure 6A–C, the tumor nodules were decreased in the CCT-β knockdown group, and reduced tumor colonies in the lungs were observed compared to those in the control group without CCT-β knockdown. Our data indicated that CCT-β knockdown decreased cancer metastasis in vivo.

### 2.7. Knocking down CCT-β Altered Cell Morphology, Decreased the Levels of EMT Markers, and Inhibited the Activity and Expression of MMP2/9

**Figure 7:**
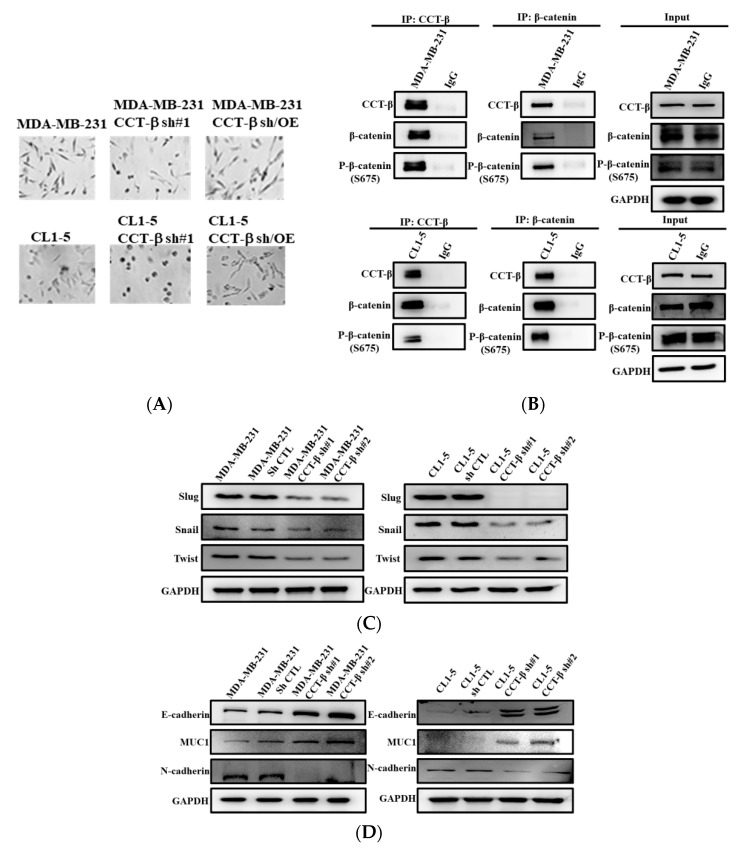

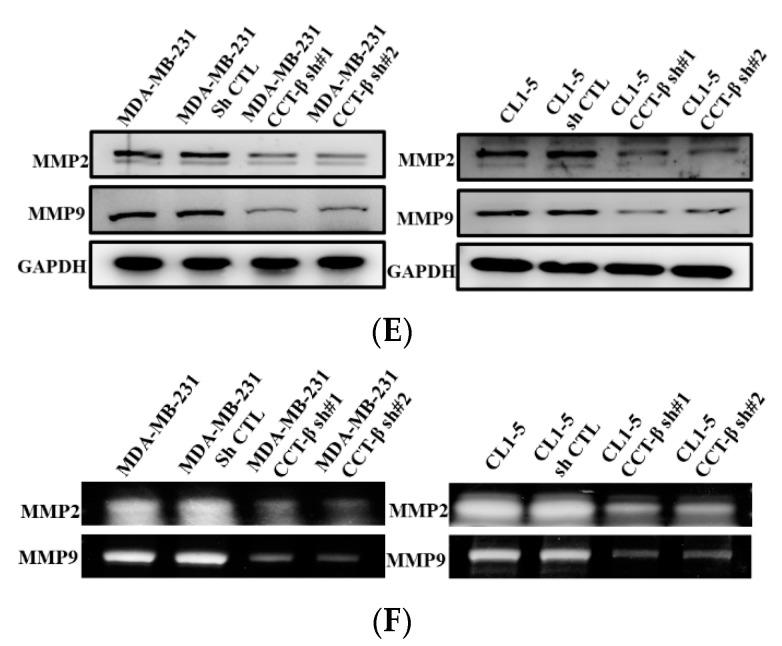
CCT-β knockdown suppressed EMT-related proteins and MMP2/9 in MDA-MB-231 and CL1-5 cells. (**A**) CCT-β knockdown and overexpression of CCT-β in CCT-β knockdown cells induced morphological changes of MET and EMT, respectively. Representative photos show the morphology of cells without or with CCT-β knockdown and overexpression of CCT-β in the CCT-β knockdown cells, respectively, captured under a microscope with a 10× objective. (**B**) Coimmunoprecipitation analysis demonstrates the physical association between CCT-β and β-catenin in MDA-MB-231 and CL1-5 cells. IgG means control, non-immune immunoglobulin. (**C**–**E**) CCT-β knockdown suppressed the expression of EMT-related proteins and MMP2/9 in MDA-MB-231 and CL1-5 cells. GAPDH was used as a loading control. (**F**) CCT-β knockdown inhibited MMP2/9 activity in MDA-MB-231 and CL1-5 cells. The conditioned media were collected and MMP2/9 activities were determined by gelatin zymography. The activities of these proteases were subsequently quantified by densitometric analysis.

MDA-MB-231 and CL1-5 cells are generally dispersed with reduced cell-cell contact and exhibit an elongated, fibroblast-like morphology. However, after silencing of CCT-β, MDA-MB-231 cells appeared cuboidal in shape, losing their apicobasal polarity and showing a morphology reminiscent of cells that have undergone mesenchymal-to-epithelial transitions (MET) (Figure 7A), the reverse process of EMT. Morphological changes in CL1-5 cells were similar to those in MDA-MB-213 cells after silencing CCT-β. Overexpression of CCT-β in the CCT-β knockdown MDA-MB-231 and CL1-5 cells recovered the fibroblast-like morphology (Figure 7A). To determine whether CCT-β induced EMT through β-catenin, we performed co-IP analysis using CCT-β antibody and β-catenin antibody in MDA-MB-231 and CL1-5 cells to show the association of endogenous CCT-β with β-catenin and a non-degradable form of S675-phosphorylated β-catenin (Figure 7B). This pS675 form might be stabilized by CCT and enters into cell nuclei to overexpress EMT markers, such as zinc-finger-containing Snail family members, Slug, Snail, and basic helix-loop-helix (bHLH) factor, and Twist, which are transcription factors that upregulate the expression of tumor suppressors. We therefore analyzed the expression of these EMT regulators in the cells after CCT-β knockdown. As shown in Figure 7C, the expression levels of Snail and Slug were decreased in the CCT-β knockdown MDA-MB-231 and CL1-5 cells. The expression of Twist was decreased in both types of siRNA-treated cells. In addition to the decreased levels of transcription repressors, we also observed an increased level of the epithelial marker E-cadherin and downregulation of the mesenchymal marker N-cadherin in the CCT-β knockdown cells (Figure 7D).

Degradation of extracellular matrix (ECM) by matrix metalloproteases (MMPs), particularly MMP2/9, is required for cancer cell metastasis [23]. Therefore, we evaluated the changes in MMP2 and MMP9 expression and activity in the cancer cells by western blotting and gelatin zymography assays, respectively, under the same conditions used in the migration/invasion assays. Knocking down CCT-β induced a noticeable reduction in MMP2/9 protein expression (Figure 7E). Furthermore, gelatin zymography assays revealed a significant reduction in MMP2/9 activity in the MDA-MB-231 and CL1-5 cells upon CCT-β knockdown (Figure 7F). These results indicated that overexpressed CCT-β promoted EMT in MDA-MB-231 and CL1-5 cells.

### 2.8. Knocking down CCT-β Inhibited Metastasis by Downregulating the AKT-GSK3β-β-catenin and XIAP-Survivin Pathways

**Figure 8:**
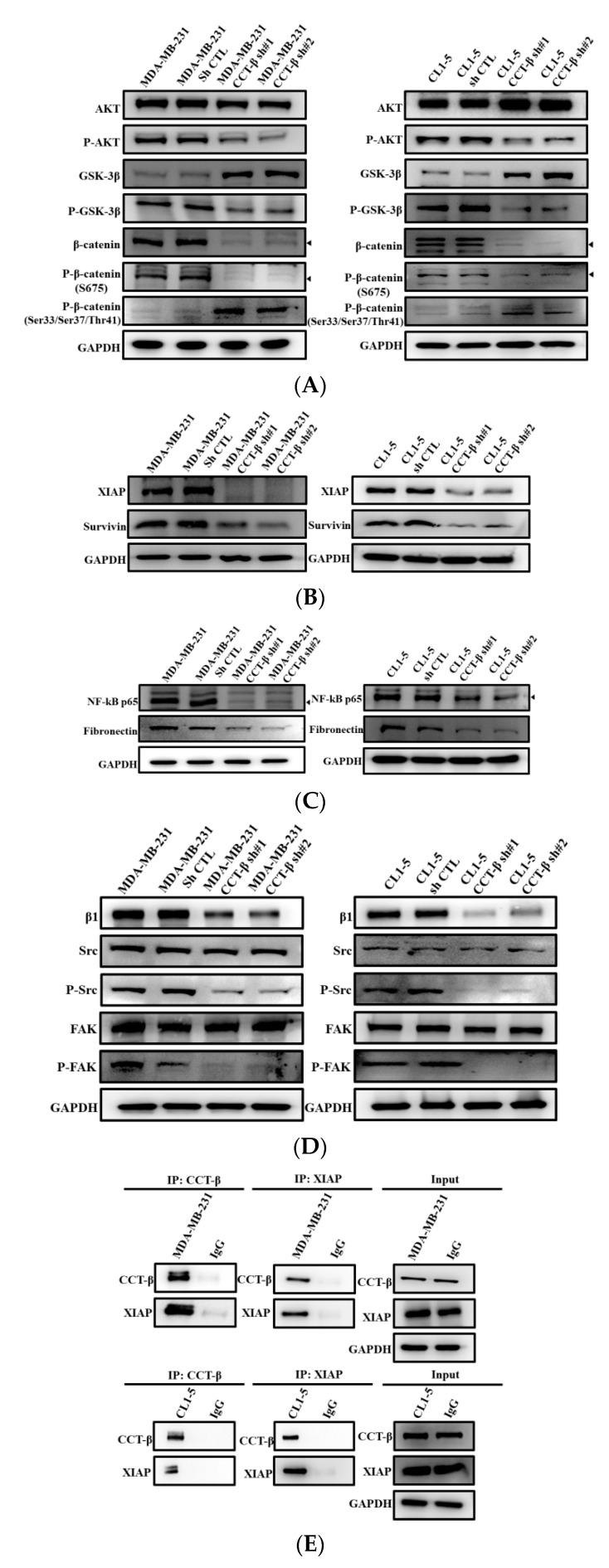
CCT-β knockdown affected the expression levels of proteins in the AKT-GSK3β-β-catenin and XIAP-Survivin signaling pathways. (**A**–**D**) CCT-β knockdown suppressed the expression of proteins in the β-catenin and XIPA-Survivin pathways. The cell lysates were collected for western blotting. GAPDH was used as a loading control. (**E**) Coimmunoprecipitation analysis revealed the physical association between CCT-β and XIAP in the MDA-MB-231 and CL1-5 cells. IgG means control, non-immune immunoglobulin.

Several studies have indicated that transcription factors (e.g., those in the AKT/β-catenin pathways) are involved in regulating the activities of MMP2 and MMP9 [24] and thus cancer cell metastasis. As shown in Figure 8A, CCT-β knockdown in MDA-MB-231 and CL1-5 cells led to decreased p-AKT and p-GSK-3β levels and increased GSK-3β levels. The results showed that CCT-β knockdown decreased Ser675-p-β-catenin, which can enter the nucleus, and increased Ser33/37-p-β-catenin, which is the degradable form (Figure 8A), causing an anti-migratory/invasive effect.

However, several studies have shown that XIAP regulates cell migration and invasion via cooperation with Survivin [25,26]. To determine whether inhibition of migration by CCT-β knockdown could be mediated through the XIAP-Survivin pathway, we utilized western blotting experiments to show that CCT-β knockdown in the MDA-MB-231 and CL1-5 cells led to decreased Survivin (Figure 8B), NF-κB, and fibronectin levels (Figure 8C), as well as integrin β1, Src, p-Src, FAK, and p-FAK levels (Figure 8D). Therefore, the metastatic behavior of MDA-MB-231 and CL1-5 cells was due to the promotion of these two pathways after overexpressing CCT-β. Direct association of endogenous CCT-β with XIAP in the MDA-MB-231 and CL1-5 cells was shown by co-IP experiments using CCT-β and XIAP antibodies (Figure 8E).

## 3. Discussion

We have demonstrated the importance of CCT-β in predicting a poor chemotherapeutic response in lung adenocarcinoma [22]. Here, we further show that the CCT-β subunit more significantly correlates with breast cancer patient survival rates than the other subunits (Figure 1A). The prognostic significance of CCT-β expression is more obvious when patients were treated with chemotherapy. These findings indicate that chemoresistance is probably an outcome of CCT-β overexpression, resulting in a lower RFS (Figure 1E). Therefore, by knocking down CCT-β in the CCT-β-overexpressing MDA-MB-231 and CL1-5 cells, the cancer cells showed increased sensitivity toward the anti-cancer drugs doxorubicin and paclitaxel (Figure 2D,E). Overexpression of CCT-β in these cancer cells enhanced chemoresistance (Figure 2H,I). Overexpressing CCT-β in MCF-7 that has an endogenously low level of CCT-β increased chemoresistance to paclitaxel (Figure 4E) and knockdown of CCT-β from 7TR reduced its chemoresistance (Figure 4H). In addition to chemoresistance, metastasis caused by CCT-β overexpression might also be responsible for the low RFS. While both MDA-MB-231 and CL1-5 cell lines are highly metastatic, knocking down CCT-β reduced the cell invasion/migration (Figure 5A–D). Overexpression of CCT-β in these CCT-β knockdown cells recovered cell invasion/migration (Figure 5E,F). In addition to the cell-based experiments, we further demonstrate that knocking down CCT-β decreased the intravasation and colonization processes in the lungs of tumor-bearing mice by performing an intracardiac injection of MDA-MB-231 cells (Figure 6).

CCT-β does not work alone but forms a complex with other subunits to perform the chaperonin function. This finding is supported by the observation that when CCT-β was knocked down, other subunits were simultaneously suppressed (Figure 2C), and when CCT-β was overexpressed, other subunits were also upregulated (Figure 2G). This raises the possibility that in fact two different regulatory mechanisms may be involved in determining CCT abundance: (1) mRNA expression levels may be regulated by transcriptional mechanisms (as reflected in the K-M Plotter data) for each subunit individually, and (2) insufficient CCT-β protein levels may lead to the degradation of the other subunits, precluding the formation of the CCT complex. In addition, it is also conceivable that such a limiting effect exists also for the other subunits, and it is the appropriate (equimolar) protein subunit mole ratio of the various subunits that determines the amount of CCT. Therefore, the importance of other subunits in the functions of CCT cannot be excluded.

**Figure 9:**
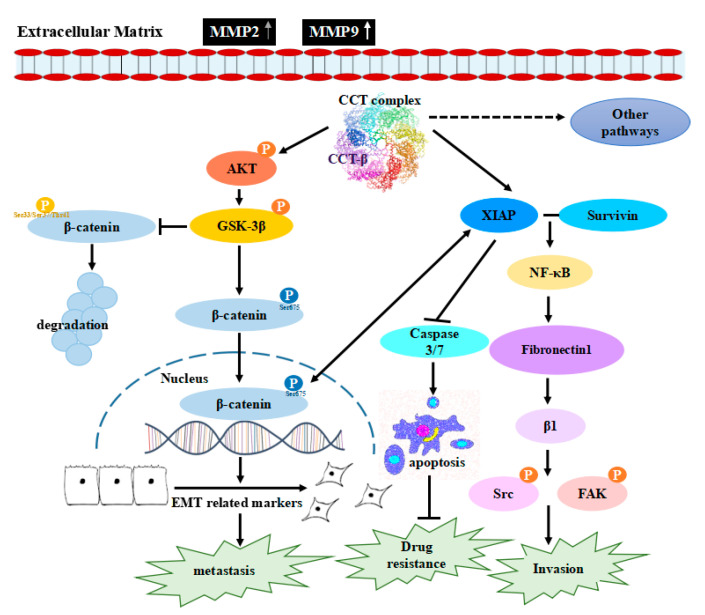
The molecular mechanism of CCT-β overexpression-induced chemoresistance, cell migration and invasion. CCT promotes the AKT-GSK3β-β-catenin and XIAP-Survivin signaling pathways by directly interacting with β-catenin and XIAP, leading to increased drug resistance, cell migration and invasion.

Our mechanistic studies reveal that overexpressed CCT-β increased the level of the CCT complex to stabilize β-catenin and XIAP (Figure 3 and Figure 4), enhancing chemoresistance/metastasis through the AKT-GSK3β-β-catenin and XIAP-Survivin pathways (Figure 8). Although we show that CCT-β knockdown did not affect the cell survival (Figure S7A,B) and cell cycle (Figure S7C,D), overload/misfolding of other proteins might have an effect on chemoresistance and other malignancy-related phenomena due to the absence of sufficient amounts of the CCT complex; thus, the other regulatory pathways likely exist (Figure 9).

For chemoresistance, in the XIAP-Survivin pathway as shown in Figure 9, XIAP is an anti-apoptotic protein known to inhibit caspases [27,28,29], and thus, its antagonists exhibit broad antitumor activities [30,31,32]. FOXM1 can also target XIAP and Survivin to modulate breast cancer survival and chemoresistance [33]. Between the two pathways in Figure 9, XIAP interacts with GSK3 in mammalian cells to inhibit intrinsic apoptotic signaling by GSK3 [34]. XIAP could also promote monoubiquitination of the corepressor Groucho/TLE family proteins, decreasing their affinity with the TCF/Lef family of transcription factors and allowing assembly of transcriptionally active β-catenin-TCF/Lef complexes [35,36]. Moreover, studies have shown that β-catenin promotes drug resistance by coexpressing XIAP and P-glycoprotein, a drug pump [37]. We show in this study that CCT-β overexpression increases both β-catenin and XIAP expression in MDA-MB-231 and CL1-5 cells (Figure 3C,E), which do not have a P-glycoprotein (we experimentally checked the cells with antibodies against P glycoprotein and other multidrug transporters, but found absence of these transporters, data not shown). In the β-catenin pathway, CCT-β knockdown reduces the levels of p-AKT and p-GSK3β and thus the nuclear level of p-β-catenin. Related Wnt/β-catenin signaling has been associated with chemoresistance in cancer cells [38,39,40,41] and is essential for lung tumorigenesis as well as maintenance of stemness characteristics in multiple cancer subtypes, including NSCLC [42,43]. Here, we show that activation of β-catenin could be mediated through CCT-β overexpression.

For metastasis, reduced levels of Survivin, NF-κB, fibronectin, p-Src, and p-FAK were observed when CCT-β was knocked down (Figure 8), consistent with the finding that overexpressed CCT-β upregulates the XIAP-Survivin signaling pathway. It has been reported that the XIAP-Survivin complex could activate NF-κB, which in turn leads to increased fibronectin gene expression, signaling by β1 integrin, and activation of the cell motility kinases FAK and Src [25]. Survivin is a known downstream target of the Wnt/β-catenin pathway [44,45]. Inhibitors of apoptosis (IAPs), a family of anti-apoptotic proteins to which XIAP belongs, could be also regulators of cell migration and development [46]. As reported here, being stabilized by CCT, XIAP enhances metastasis through the XIAP-Survivin signaling pathway (Figure 9).

Dysregulation of the Wnt/β-catenin signaling pathway leads to uncontrolled tumor cell proliferation, metastasis, and resistance to apoptosis in lung cancer and breast cancer [47,48,49]. Wnt/β-catenin signaling can combine with other oncogenic pathways in the lung epithelium to produce a more aggressive tumor phenotype by inducing an embryonic distal progenitor phenotype and by decreasing E-cadherin expression [50]. Wnt/β-catenin signaling has been implicated in mammary oncogenesis [51] and in a subgroup of invasive breast cancers of TNBC, which is associated with a poor clinical outcome [52,53]. Instead of being triggered by Wnt, enhanced levels of p-AKT, p-GSK3β, and the nuclear p-β-catenin were observed after overexpression of CCT-β; these results show the role of CCT-β in promoting β-catenin function, leading to upregulation of EMT markers and MMP2/9, thus increasing metastasis.

Taken together, our findings suggest the critical roles of CCT, particularly CCT-β, in conferring tumors with a chemoresistant and metastatic phenotype through the signaling pathways (Figure 9). Our studies also offer a new chemotherapeutic strategy to treat CCT-β-overexpressing tumors by destabilizing the interactions of CCT with its clients. We have developed a PPI inhibitor to disrupt the complex of CCT-β with β-tubulin and selectively kill CCT-β-overexpressing cancers that are highly resistant to anticancer drugs [22]. A cytotoxic peptide was found to target CCT and overexpressed CCT-β enhanced breast cancer susceptibility to this peptide [54]. Other PPI inhibitors could be developed to target the interactions of CCT with proteins related to chemoresistance and metastasis for cancer therapy to overcome these hurdles.

## 4. Materials and Methods

### 4.1. Reagents

Most chemicals used and Protein A-agarose were purchased from Sigma-Aldrich (St. Louis, MO, USA). The antibody for CCT-β was purchased from Abcam (Cambridge, MA, USA) for the co-IP assay. For western blots, the specific antibodies for GAPDH (100118), CCT-α (16399), CCT-β (112283), CCT-γ (33073), CCT-δ (33536), CCT-ε (110167), CCT-ζ (105148), CCT-η (101347), CCT-θ (115466), XIAP (111202), β-catenin (101435), P-β-catenin (S675) (132611), P-β-catenin (Ser33/Ser37/Thr41) (132605), Slug (121924), Snail (100754), Twist (127310), E-cadherin (100443), MUC1 (100459), N-cadherin (112734), MMP2 (104577), MMP9 (100458), AKT (121973), P-AKT (128414), GSK-3β (111192), P-GSK-3β (50090), Survivin (100052), NF-kB p65 (102090), fibronectin (112794), β1 (112971), Src (134412), P-Src (54701), FAK (50489), and P-FAK (129840) were from GeneTex (Irvine, CA, USA). The catalog numbers are in parentheses.

### 4.2. Collection of Clinical Samples and Immunohistochemistry

The breast cancer tissues used were from the Taipei Medical University (TMU)-managed Wan Fang Hospital. Patient information, including gender, age, and histopathological diagnoses, was collected. The study was carried out with the approval of the Institutional Review Boards and with permission from the ethics committees of the institution involved (TMU-IRB 99049). For IHC staining, the paraffin-embedded tumor sections (3 μm thickness) of tissue microarrays prepared in house according to the literature [55] were deparaffinized using xylene and rehydrated in a graded series of ethanol with a final wash in tap water. Antigen retrieval was performed with Target Retrieval Solution (DAKO) in a Decloaking Chamber (Biocare Medical, Pacheco, CA, USA). Endogenous peroxidase activity was quenched by hydrogen peroxide. The sections were then incubated with CCT-β antibody (Santa Cruz Biotechnology, Dallas, TX, USA) at 4 °C overnight in a humid chamber. A Vectastain ABC peroxidase system (Vector Laboratories, Burlingame, CA, USA) was used to detect the reaction products. A four-point staining intensity scoring system was devised for determining the relative expression of CCT-β in cancer specimens; the staining intensity score ranged from 0 (no expression) to 3 (maximal expression). All of the IHC staining results were reviewed and scored independently by two pathologists.

### 4.3. Cell Culture

A549, ZR-75-1, MDA-MB-231, and MCF-7 cells were obtained from the Bioresource Collection and Research Centre (BCRC) in Hsinchu, Taiwan. The paclitaxel-resistant 7TR cells were generated by treating MCF-7 with increased concentrations (0, 5, 10, 20, 40, 60, 80, 100, 120, 140, 160, 180, 200, 220, 240, 260, 280, and 300 nM) of paclitaxel continuously and selecting the survived cells. HEK-293T was a gift from Dr. Shih-Hsiung Wu (Institute of Biological Chemistry, Academia Sinica, Taipei, Taiwan). The human lung adenocarcinoma cell lines with less invasive (CL1-0) and highly invasive capacities (CL1-5) were established previously [56]. A549 cells were cultivated in F-12K, MCF-7 and 7TR in MEM, ZR-75-1, CL1-0, and CL1-5 in RPMI-1640, MDA-MB231 and HEK-293T in DMEM. Each medium was supplemented with 10% fetal bovine serum (FBS) that was heat-inactivated at 56 °C for 30 min in a water bath and 1% of a commercial product [penicillin-streptomycin-Glutamine (100×)] (Gibco catalog number: 10378016). All cell culture media and supplements were purchased from Gibco (Carlsbad, CA, USA). The cells were maintained in the presence of 5% CO_2_ at 37 °C.

### 4.4. Plasmids and Cell Transfection

Two CCT-β knockdown plasmids (TRCN0000029499 containing shRNA 5′-CCGGGCCTCTCTTATGGTAACCAATCTCGAGATTGGTTACCATAAGAGAGGCTTTTT-3′ and TRCN0000285418 containing shRNA 5′-CCGGGTTTATCGGTGCCCATTATATCTCGAGAT ATAATGGGCACCGATAAACTTTTTG-3′) were obtained from the RNA Technology Platform and Gene Manipulation core (RNAi core) in Taipei, Taiwan. Each shRNA-expressing virus was prepared by cotransfection of the plasmid containing a shRNA cloned with the pLAS3w_Ppuro vector, and the two plasmids, CMV-8.91 and pMD.G, for producing virus, into HEK-293T cells using TurboFect transfection reagent (Thermo Fisher Scientific Co., Fair Lawn, NJ, USA) according to the procedure provided by the RNAi core. Cultures of virus-infected MDA-MB-231 and CL1-5 cells were selected with puromycin antibiotic for knockdown strains.

For overexpressing CCT-β, the forward primer 5′-CGCGGCTAGCATGGCGTCCCTTTCCCTT-3′ and the reverse primer 5′-CGTAGAATTCTTAAGCGTAATCTGGAACATCGTATGGGTAA CAGGGGTGGTGATCAGG-3′ were used for PCR amplification of the gene. Total RNA extracts from MDA-MB-231 and CL1-5 cells were reverse-transcribed into cDNA using the GScript First-Strand Synthesis Kit (GeneDireX, Taipei, Taiwan); this molecule was used as the template for PCR. cDNA was amplified by PrimeSTAR GXL DNA polymerase (TaKaRa Bio USA, Inc., Mountain View, CA, USA). The CCT-β-encoding gene was cloned with pLAS5w_Pbsd vector and cotransfected with CMV-8.91 and pMD.G plasmids into HEK-293T cells for producing virus as described above. For selecting CCT-β overexpressed strains, an antibiotic blasticidin S was used.

### 4.5. Western Blot Analysis

The cell lysates containing 60 μg proteins were boiled for 5 min in SDS sample buffer (62.5 mM Tris-HCl pH 6.7, 1.25% SDS, 12.5% glycerol, and 2.5% β-mercaptoethanol). The SDS-polyacrylamide gels were homemade by preparing 8% separating gel solution using 46% of ddH_2_O, 26% of 30% acrylamide stock solution, 26% of 1.5 M Tris (pH 8.8), 1% of 10% SDS stock solution, 1% of 10% ammonium persulfate stock solution, and 0.06% Tetramethylethylenediamine (TEMED), and the stacking gel solution using 68% of ddH_2_O, 16.6% of 30% acrylamide stock solution, 12.6% of 1.0 M Tris (pH 6.8), 1% of 10% SDS stock solution, 1% of 10% ammonium persulfate stock solution, and 0.1% of TEMED. 10% and 12% separating gel solutions were prepared by 34% and 40% of 30% acrylamide stock solution, respectively. For western blotting, proteins were transferred onto a PVDF membrane (Merck Millipore, Darmstadt, Germany) and the membrane was incubated with the specific antibodies as described in Section 4.1. Primary antibodies were diluted by 1000-fold in Tris buffered saline (TBST) buffer [20 mM Tris-base (pH 7.5), 150 mM NaCl, and 0.1% Tween 20] containing 2.5% (final concentration) non-fat milk for overnight incubation. The next day, the solution was discarded and each membrane was rinsed with 1xTBST buffer for 10 min three times. Then, the horseradish peroxidase (HRP)-conjugated secondary antibody diluted 1000-fold was added and incubated for 2 h. Subsequently, the solution was discarded and each membrane was rinsed with 1xTBST buffer for 10 min three times. Then, the immunoreactive protein bands were revealed by using the enhanced chemiluminescence (ECL) system (Amersham Bioscience, Tokyo, Japan). All the uncropped western blots are shown in Figure S8.

### 4.6. Coimmunoprecipitation Assay

Antibodies against CCT-β, β-catenin, XIAP, and IgG were added to MDA-MB-231 and CL1-5 cell lysates in immunoprecipitation buffer (25 mM Tris-HCL, pH 7.6, 200 mM NaCl, 0.1% NP-40, 6% glycerol, 1 mM EDTA, and 0.5 mM DTT), and the solutions were rotated at a speed of 25 rpm at 4 °C. The next day, Protein A-agarose was added and the solutions were rotated again. After 1 h, the solutions were washed with immunoprecipitation buffer and centrifuged. After the supernatant was removed, the precipitate was added to SDS sample buffer (62.5 mM Tris-HCl, pH 6.7, 1.25% SDS, 12.5% glycerol, and 2.5% β-mercaptoethanol) and boiled at 100 °C for 10 min for SDS-PAGE analysis.

### 4.7. Flow Cytometry Analysis

Cells (5 × 10^5^) were fixed in 70% ice-cold EtOH for 30 min at RT. The cells were washed with PBS once and then incubated with propidium iodide (PI) staining solution (PBS containing 0.1% BSA, 0.1% RNase A, and 20 ng/mL PI) for 30 min at RT in the dark. After incubation, the cells were analyzed by flow cytometry to assess the DNA content.

### 4.8. MTT Assay

Cell viability was determined using a 3-(4,5-dimethylthiazole-2-yl)-2,5-diphenyl tetrazolium bromide (MTT) assay. MDA-MB-231 and CL1-5 cells (5 × 10^3^ cells per well) with knockdown or overexpression of CCT-β were seeded in 96-well plates for 24 h and then treated with various concentrations of doxorubicin for another 72 h. Subsequently, 20 μL of MTT solution (5 mg/mL) was added to each well and the mixture was incubated for an additional 3 h. The formazan precipitate was dissolved in 100 μL of dimethyl sulfoxide without eliminating the water phase by centrifugation, and the solution was vigorously mixed to dissolve the reacted dye. After 2 h, the absorbance of each well was read at 590 nm with a spectrophotometer.

### 4.9. Wound-Healing Assay

For the wound-healing assay, cells were seeded in 24-well plates in culture medium and transfected with either siRNA or plasmids. The cells (2 × 10^5^ cells/mL) were starved with serum-free medium overnight and incubated with 5 μg/mL mitomycin C (Target Molecular Corp., Boston, MA, USA) for 2 h prior to the assay. A scratch was drawn at the center of the well, and then, the wounded monolayer was photographed at the indicated time intervals via an Eclipse TS100 inverted microscope (Nikon, Tokyo, Japan). For quantification of the migrated cells, pictures of the initially wounded monolayers were compared with the corresponding pictures of cells at the end of incubation. Artificial lines fitting the cutting edges were drawn on pictures of the original wounds and overlaid on the pictures of the cultures after incubation. Cells that had migrated across the red lines were counted under a microscope at a magnification of 4×.

### 4.10. Matrigel Invasion Assay

Cells were analyzed for invasion with a transwell assay. In brief, transwell devices with 8 μm pore size polyethylene terephthalate (PET) membranes (Merck Millipore, Darmstadt, Germany) were coated with 100 μL of Matrigel (300 μg/mL) (Corning, Corning, NY, USA). Cells (2 × 10^4^ cells/mL) without and with CCT-β knockdown or overexpression were suspended in 200 μL of serum-free medium, placed in the upper transwell chamber, and incubated for 24 h at 37 °C. Then, the cells on the upper surface of the filter were completely wiped away with a cotton swab, and the lower surface of the filter was fixed in methanol, stained with crystal violet, and counted under a microscope at a magnification of 10×.

### 4.11. Animal Studies

NOD/SCID mice were obtained from the National Laboratory Animal Center in Taiwan and were maintained in compliance with institutional guidelines. All animal procedures were approved by the Institutional Animal Care and Use Committee (approval number: IACUC 11-07-201) at Academia Sinica, Taiwan. For the in vivo metastatic colonization assay, cells were implanted through tail vein injection at a concentration of 1 × 10^6^ cells/100 μL PBS. The mice were humanely killed at the endpoint of experiments, and the lungs were obtained for further histological analysis.

### 4.12. Gelatin Zymography

8 × 10^5^ MDA-MB-231 and CL1-5 cells with or without CCT-β knockdown were cultivated in 10 mL of DMEM and RPMI-1640, respectively. Next day (after 12–15 h), the medium was discarded and the cells were added with 10 mL serum-free medium for each plate. After a 24 h incubation for secreting MMPs, the mixture pooled from three plates of cultures (totally 30 mL) was centrifuged at 3500 rpm to 1 mL by using a 50-mL 10K filter (Merck Millipore, Darmstadt, Germany) to concentrate the secreted MMPs. Then the total proteins were quantitated by Bio-Rad assay and the same amount (40 μg in 10 μL) of total proteins was loaded onto a 8% SDS-PAGE gel containing 0.1% gelatin to assess the enzymatic activity of MMP2/9. The gel was washed twice at RT by a wash solution containing 2.5% (*v/v*) Triton X-100 in ddH_2_O and subsequently transferred to a reaction buffer (0.02% NaN_3_, 5 mM CaCl_2_, and 50 mM Tris-HCl, pH 7.5) for enzymatic reactions at 37 °C with shaking overnight (12–15 h). Finally, the MMP gel was stained for 10 min with RapidStain (Bioman, Taipei, Taiwan) and destained in deionized water. MMPs in the loaded supernatants were observed as gelatinase-positive bands that appeared white on a blue background [24].

### 4.13. Statistical Analysis

The results are presented as the mean ± SEM. Statistical analysis was performed using the nonparametric Mann-Whitney test.

## 5. Conclusions

Endogenously high expression of the subunit CCT-β and thus CCT chaperonin might stabilize the anti-apoptotic protein XIAP and the transcription factor β-catenin. XIAP and Survivin are known to inhibit apoptosis, causing chemoresistance. Survivin, NF-κB, fibronectin, p-Src, and p-FAK are involved in metastasis. β-catenin plays roles in both chemoresistance by expressing XIAP and metastasis by upregulating EMT markers and MMP2/9. Through the AKT-GSK3β-β-catenin and XIAP-Survivin pathways promoted by CCT-β, cancer cells could gain chemoresistance and metastasis. In conclusion, understanding the mechanisms of these major obstacles for current cancer therapy could lead to useful therapeutic strategies against chemoresistant/metastatic cancers.

## Supplementary Materials

The following are available online at https://www.mdpi.com/2072-6694/12/12/3865/s1, Figure S1: Prognostic significance of various CCT subunits in breast cancer patients analyzed using the recurrence-free survival (RFS) condition in the K-M Plotter database, Figure S2: Survival analysis with a signature derived from the mean expression of all CCT subunits, Figure S3: Survival analysis against PERK, an ER stress-related protein, by using K-M Plotter database for breast cancer (gene chip), Figure S4: CCTβ upregulation associates with a poorer therapeutic response in breast cancer patients, Figure S5: Univariate and multivariate analyses under the condition of RFS against CCT-β protein expression in breast cancer patients, Figure S6: CCT-β levels vs. RFS probability analyzed by Kaplan-Meier, Figure S7. Cell survival of MDA-MB-231 and CL1-5 with or without CCT-β knockdown in the absence of drug treatment, Figure S8: Uncropped western blots. Original western blots of Figures 2, 3, 6 and 7 with intensity ratios of the bands quantitated by densitometry readings. Quantitative analyses of the proteins presented were carried out using ImageJ software and were normalized with the loading control glyceraldehyde-3-phosphate dehydrogenase (GAPDH).
