# Supplementary material for: Chaperonin-Containing TCP-1 Promotes Cancer Chemoresistance and Metastasis through the AKT-GSK3β-β-Catenin and XIAP-Survivin Pathways
Source: Cancers (Basel). 2020 Dec 21;12(12):3865. doi: 10.3390/cancers12123865 (PMC7767469; doi:10.3390/cancers12123865)
Supplement: Supplementary file 1 [file cancers-12-03865-s001.pdf]

cancers

Supplementary Materials: Chaperonin-Containing TCP-1 Promotes Cancer Chemoresistance and Metastasis through the AKT-GSK3β-β-catenin and XIAP-Survivin Pathways

Yun-Xun Chang, Yuan-Feng Lin, Chi-Long Chen, Ming-Shyan Huang, Michael Hsiao and Po-Huang Liang

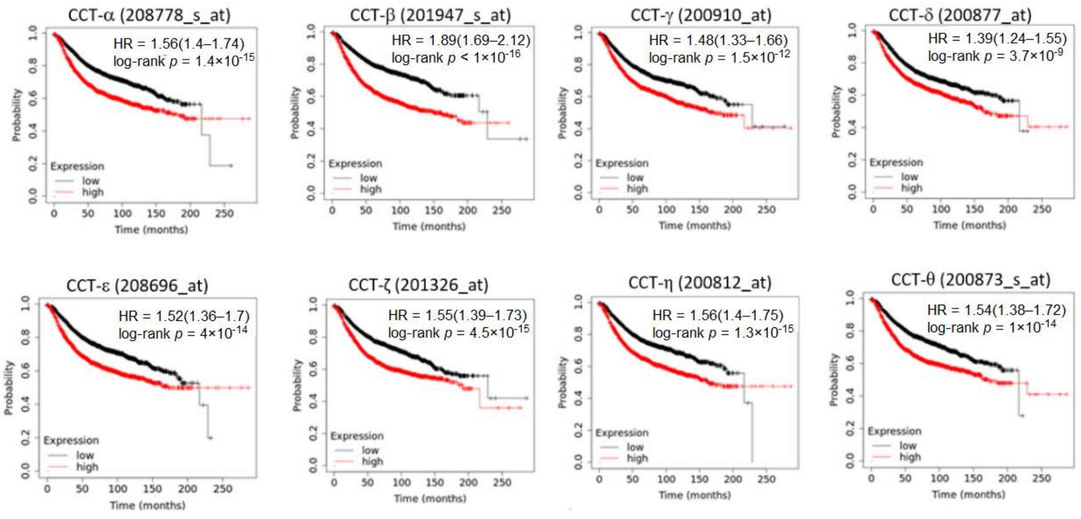

Figure S1. Prognostic significance of various CCT subunits in breast cancer patients analyzed using the recurrence-free survival (RFS) condition in the K-M Plotter database (http://kmplot.com/analysis/index.php?p=service&cancer=breast). Breast cancer patients (n = 3951) were stratified into low- and high-expression groups by the median CCT mRNA levels detected by their specific probes (shown at the top of each panel), the gene chip (Affymetrix microarray) data. The patients were not stratified with subtype and treatment specifications, the default settings in the website.

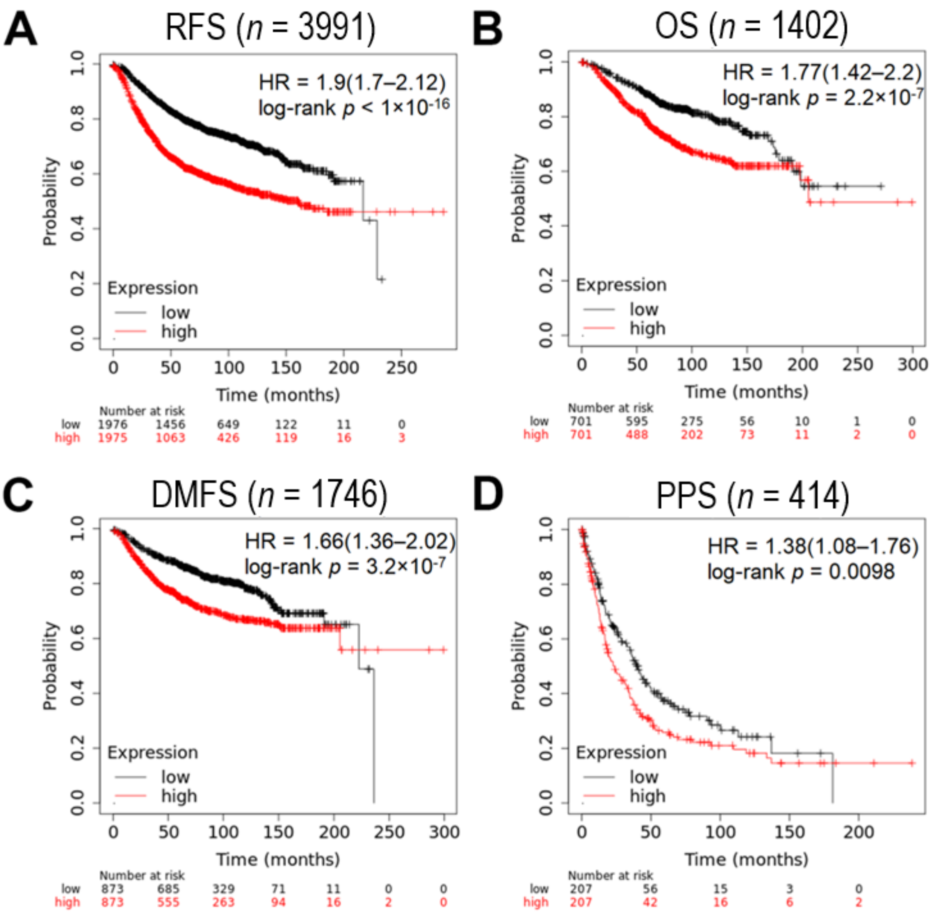

Figure S2. Survival analysis with a signature derived from the mean expression of all CCT subunits by selecting (**A**) recurrence-free survival (RFS), (**B**) overall survival (OS), (**C**) distant metastasis-free survival (DMFS), or (**D**) palliative performance scale (PPS) conditions in the K-M Plotter database (http://kmplot.com/analysis/index.php?p=service&cancer=breast). The numbers of patients analyzed are shown at the top of each Figure. The data revealed that a higher level of the signature is significantly correlated with poorer prognoses in RFS, OS, DMFS and PPS conditions. Particularly in RFS condition, a higher level of the signature refers to a highest hazard ratio (HR) in the univariate analysis, indicating a critical role of CCT subunits in the mechanism for tumor relapse of breast cancer.

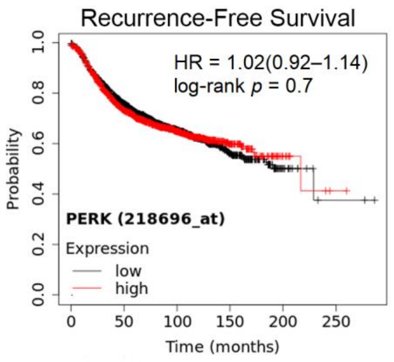

Figure S3. Survival analysis against PERK, an ER stress-related protein, by using K-M Plotter database for breast cancer (gene chip). The result shows that PERK expression does not influence recurrence-free survival condition in the detected breast cancer patients and could be a negative control of the survival analyses for eight CCT subunits.

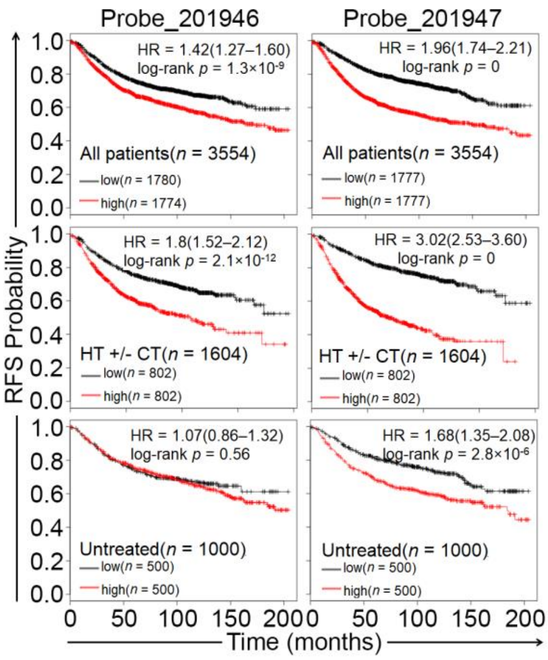

Figure S4. CCT-β upregulation associates with a poorer therapeutic response in breast cancer patients. Kaplan-Meier analyses for CCT-β gene expression detected by 2 independent probes in Affymetrix Gene Chip under the condition of recurrence-free survival (RFS) probability against breast cancer cohort from K-M Plotter database. HR, HT and CT denote hazard ratio, hormone therapy and chemotherapy, respectively.

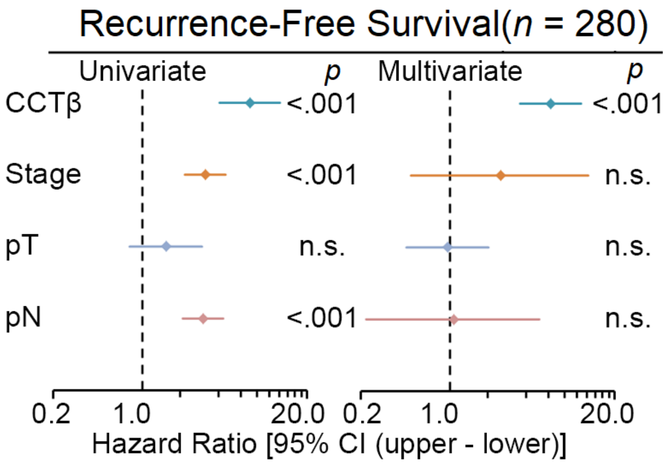

Figure S5. Univariate and multivariate analyses under the condition of RFS against CCT-β protein expression in breast cancer patients. n.s. means non-significant.

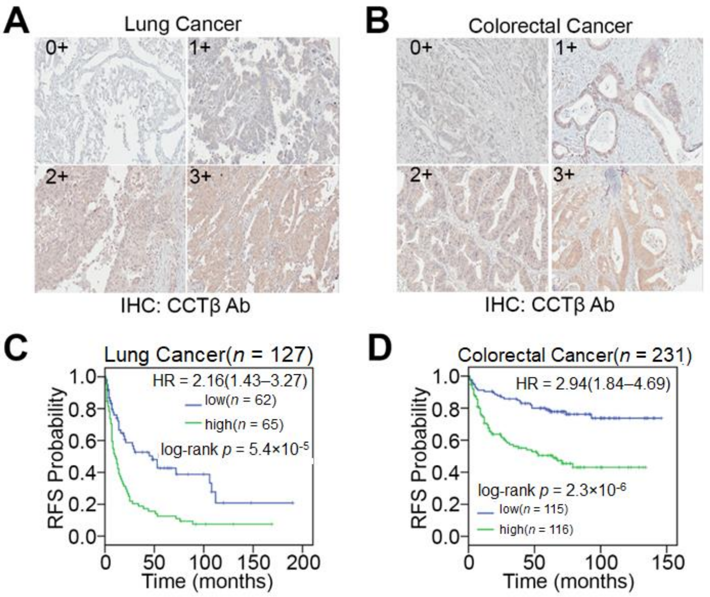

Figure S6. CCT-β levels vs. RFS probability analyzed by Kaplan-Meier. (**A, B**) IHC staining for CCT-β in lung (**A**) and colorectal (**B**) cancer tissues. The intensities of IHC staining for CCT-β protein levels were defined as strong (3+), moderate (2+), weak (1+), or undetectable (0+). (**C, D**) Kaplan-Meier analysis against CCT-β protein levels that were determined by IHC experiments under RFS conditions in clinical cohorts with lung (**C**) and colorectal (**D**) cancer.

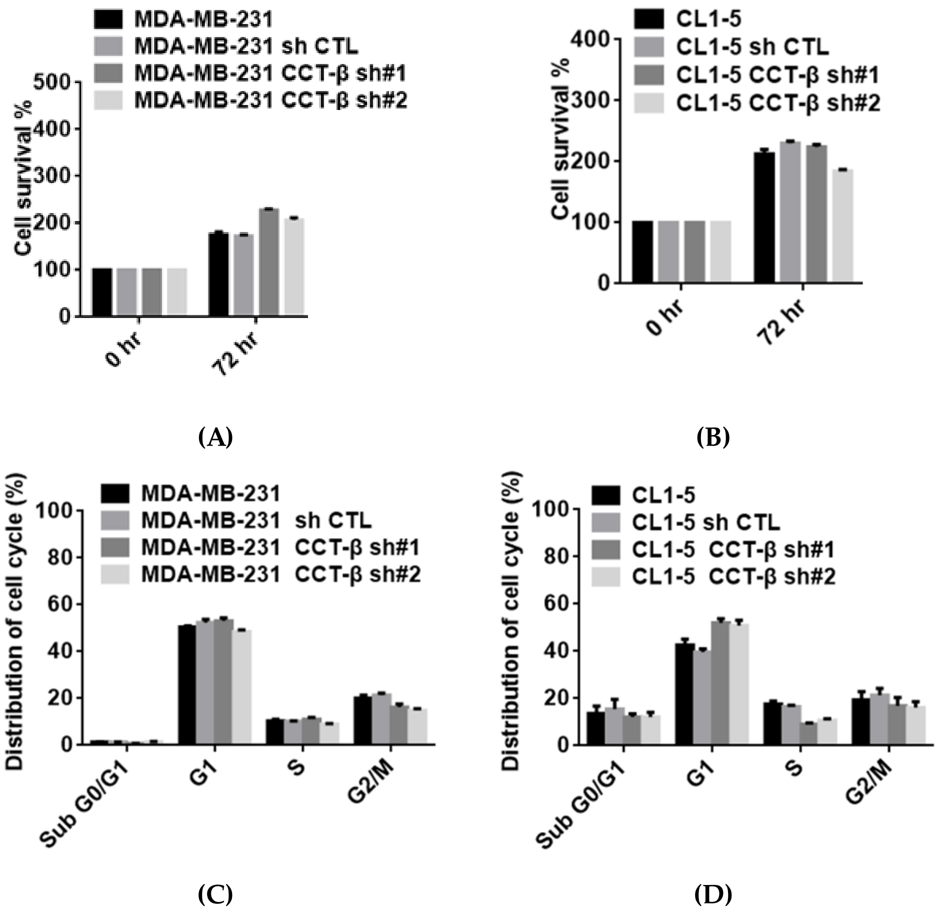

Figure S7. Cell survival of MDA-MB-231 and CL1-5 with or without CCT-β knockdown in the absence of drug treatment. (**A**) MAD-MB-231 cells, the cells with CCT-β knockdown using two

shRNAs and the vector control were grown in the medium (see Materials and Methods) and monitored for cell survival after 72 h. (**B**) CL1-5 cells, the cells with knockdown of CCT-β using two shRNAs and the vector control were grown in the medium (see Materials and Methods) and monitored for cell survival after 72 h. The data indicate no significant changes of cell survival and cell cycle by CCT-β knockdown without drug treatment. (**C**) The apoptotic levels of the MDA-MB-231 cells with and without CCT-β knockdown by flow cytometry. (**D**) The apoptotic levels of the CL1-5 cells with and without CCT-β knockdown by flow cytometry.

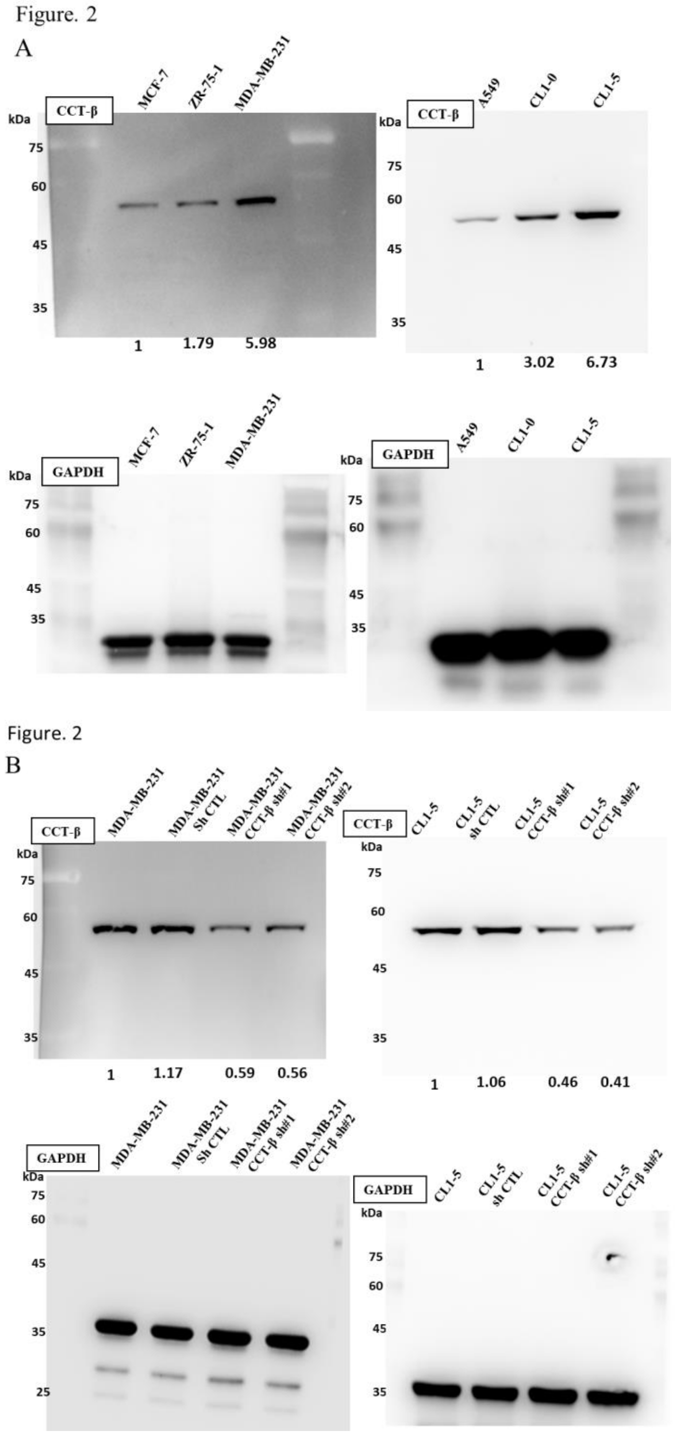

Figure. 2

C

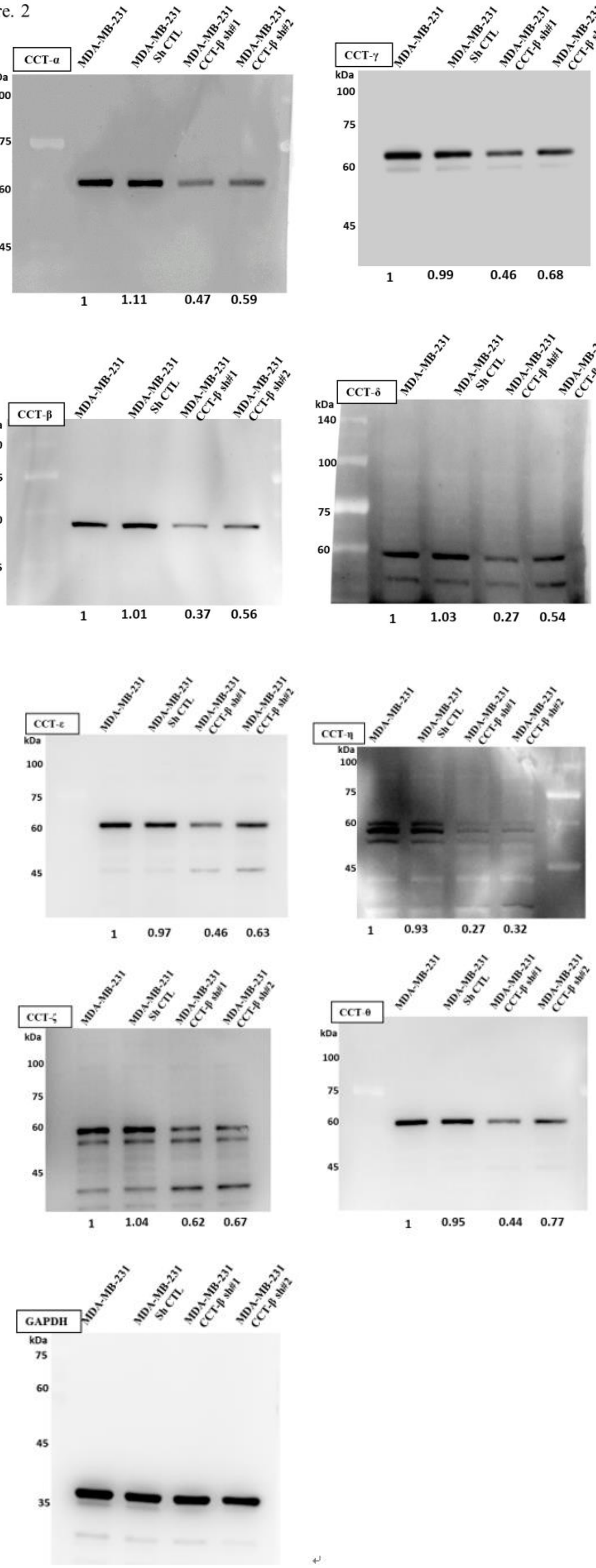
CCT-α
MDA-MB-231
MDA-MB-231 Sh CTL
MDA-MB-231 CCT-β sh#1
MDA-MB-231 CCT-β sh#2
kDa
100
75
60
45
1 1.11 0.47 0.59
CCT-γ
kDa
100
75
60
45
1 0.99 0.46 0.68
CCT-β
kDa
100
75
60
45
1 1.01 0.37 0.56
CCT-δ
kDa
140
100
75
60
1 1.03 0.27 0.54
CCT-ε
kDa
100
75
60
45
1 0.97 0.46 0.63
CCT-η
kDa
100
75
60
45
1 0.93 0.27 0.32
CCT-ζ
kDa
100
75
60
45
1 1.04 0.62 0.67
CCT-θ
kDa
100
75
60
45
1 0.95 0.44 0.77
GAPDH
kDa
75
60
45
35

Figure. 2

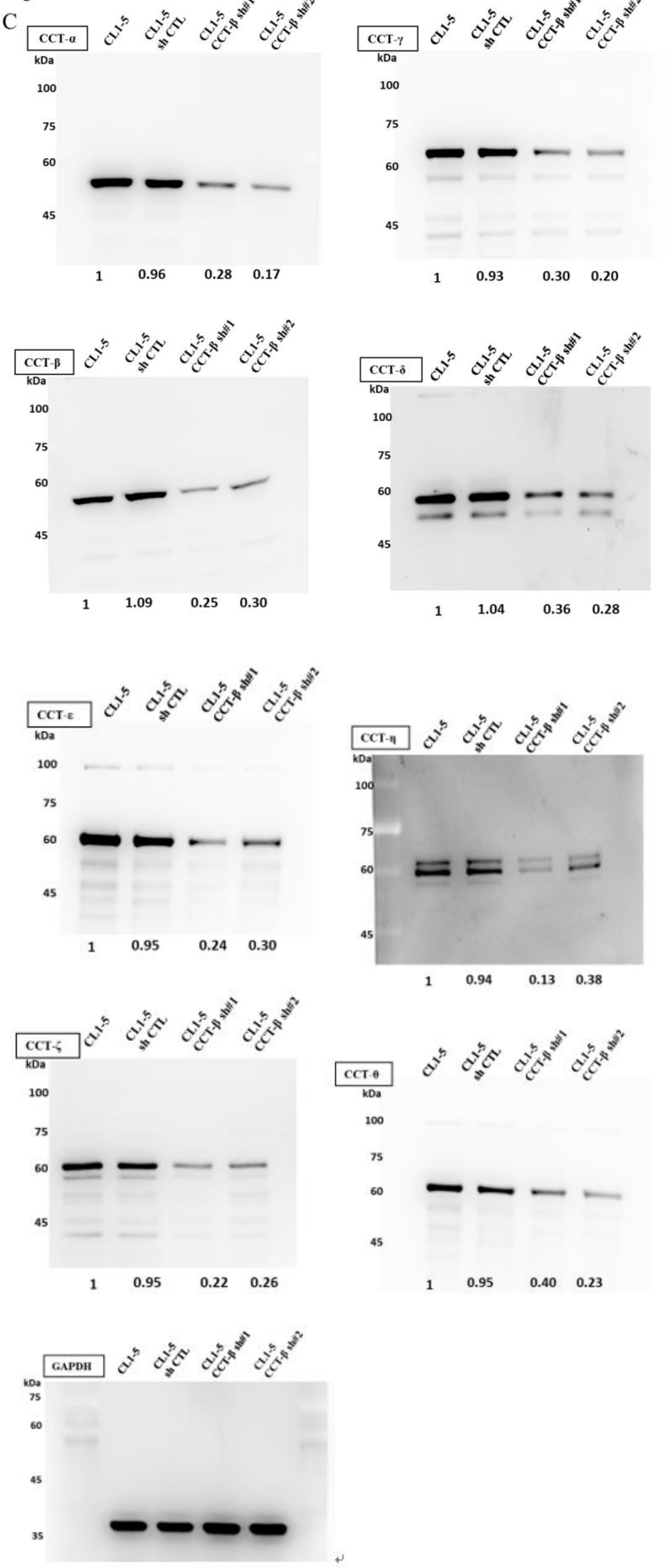
C
CCT-α
CL1-5
CL1-5 sh CTL
CL1-5 CCT-β sh#1
CL1-5 CCT-β sh#2
kDa
100
75
60
45
1 0.96 0.28 0.17
CCT-γ
1 0.93 0.30 0.20
CCT-β
1 1.09 0.25 0.30
CCT-δ
1 1.04 0.36 0.28
CCT-ε
1 0.95 0.24 0.30
CCT-η
1 0.94 0.13 0.38
CCT-ζ
1 0.95 0.22 0.26
CCT-θ
1 0.95 0.40 0.23
GAPDH
35

Figure. 2

F

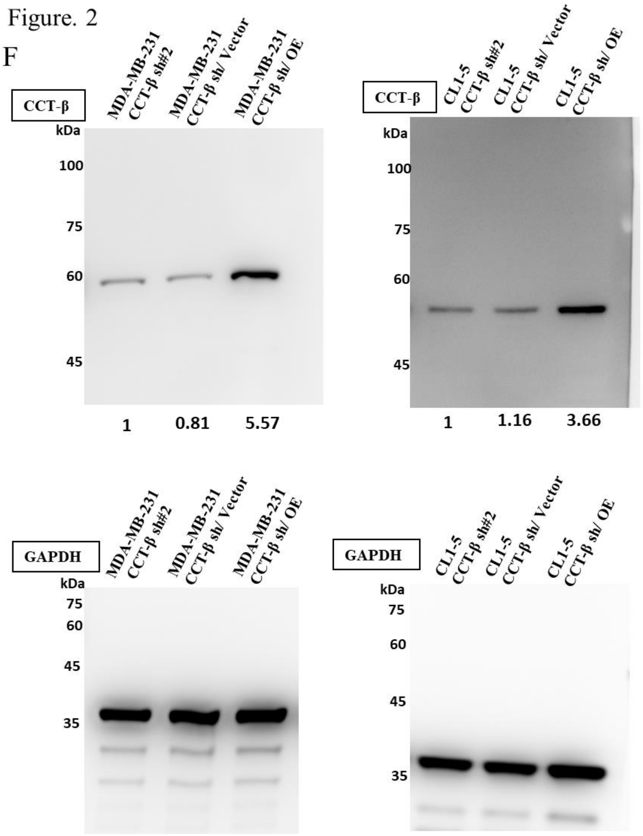

Figure. 2

G

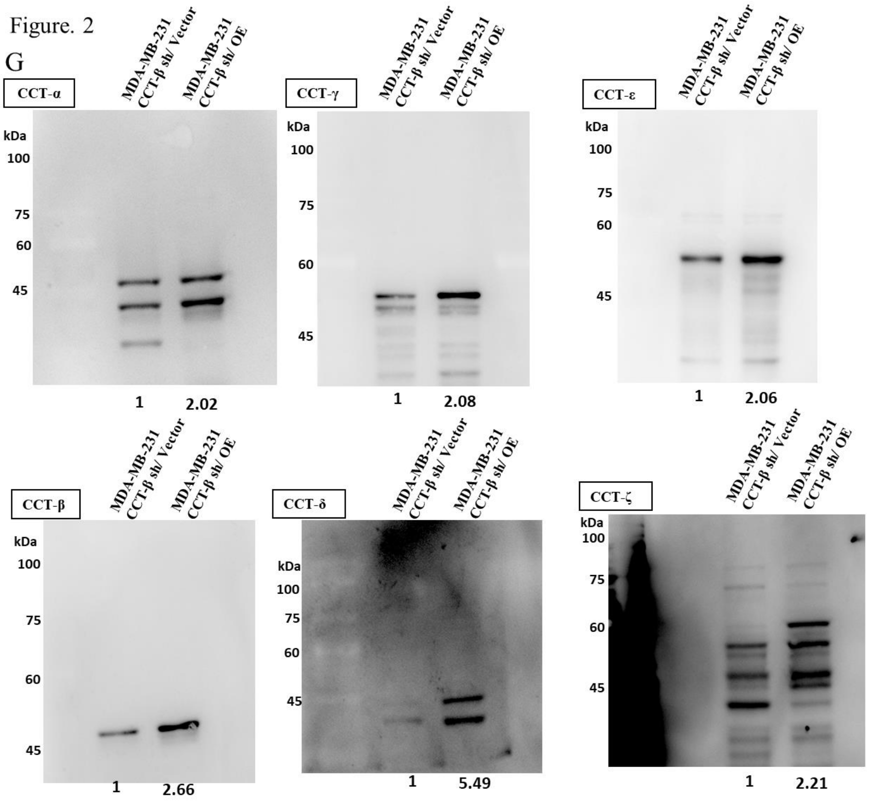

Figure. 2

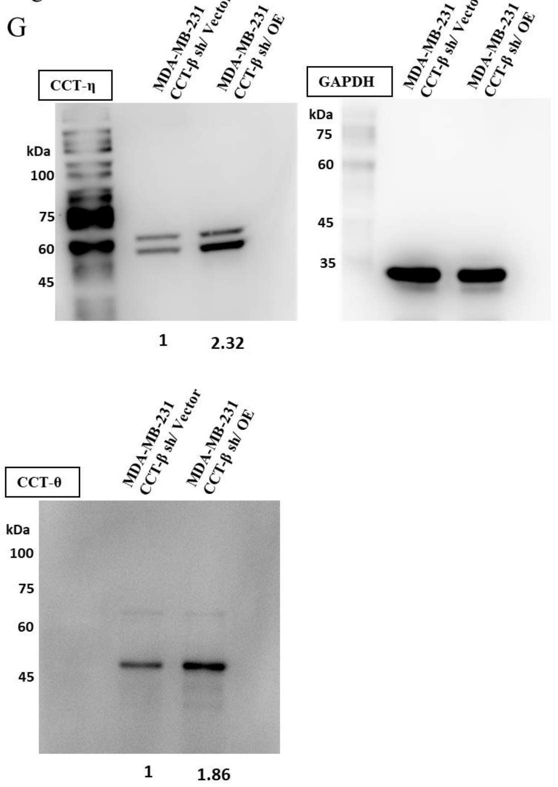

Figure. 2

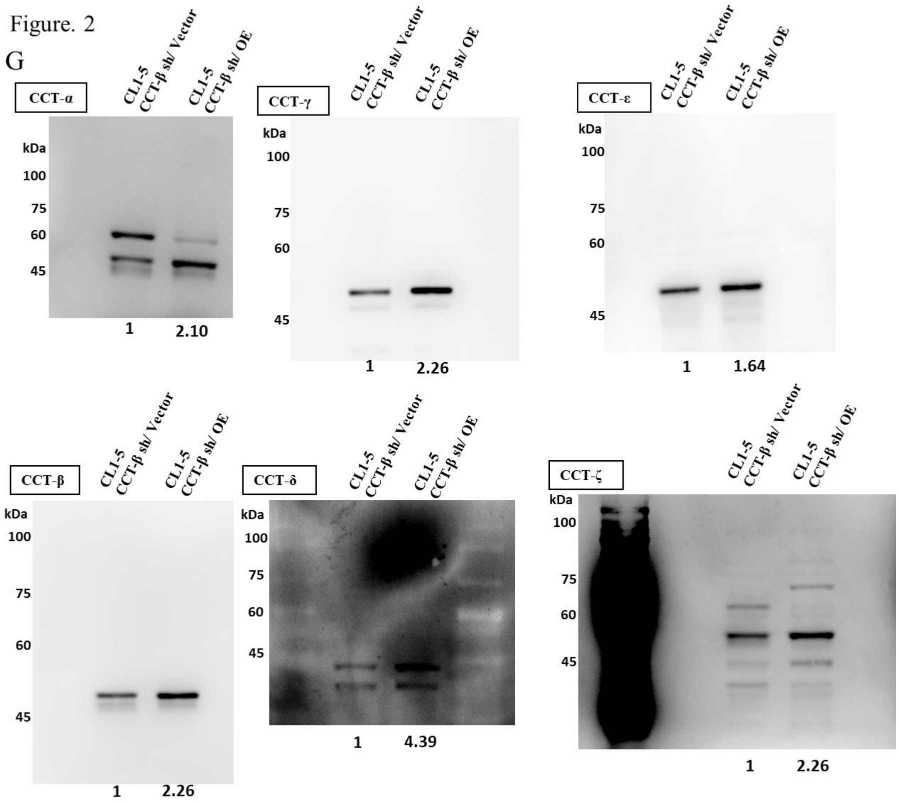

Figure. 2

G

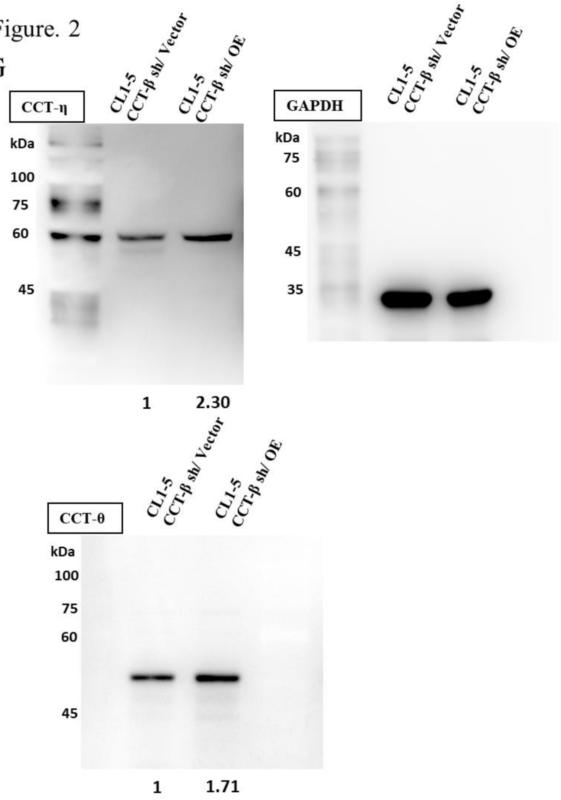

Figure. 3

A

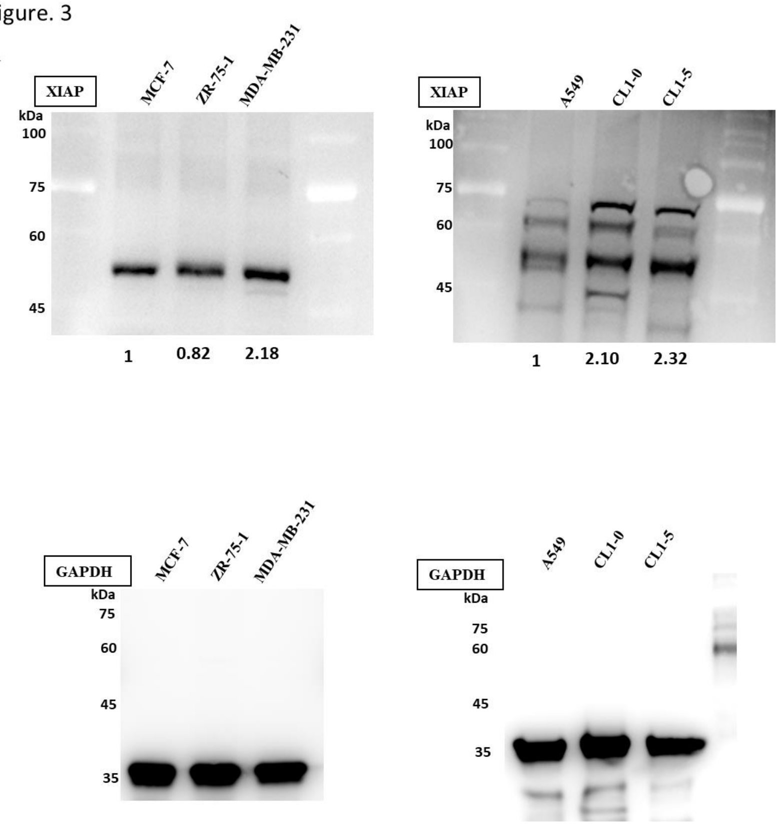

Figure. 3
B

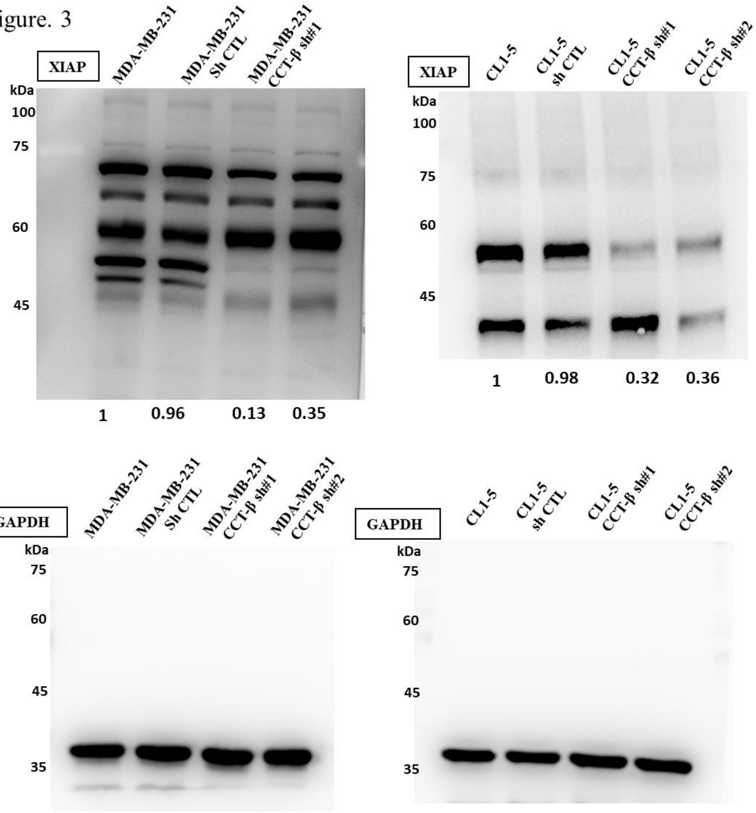

Figure. 3
C

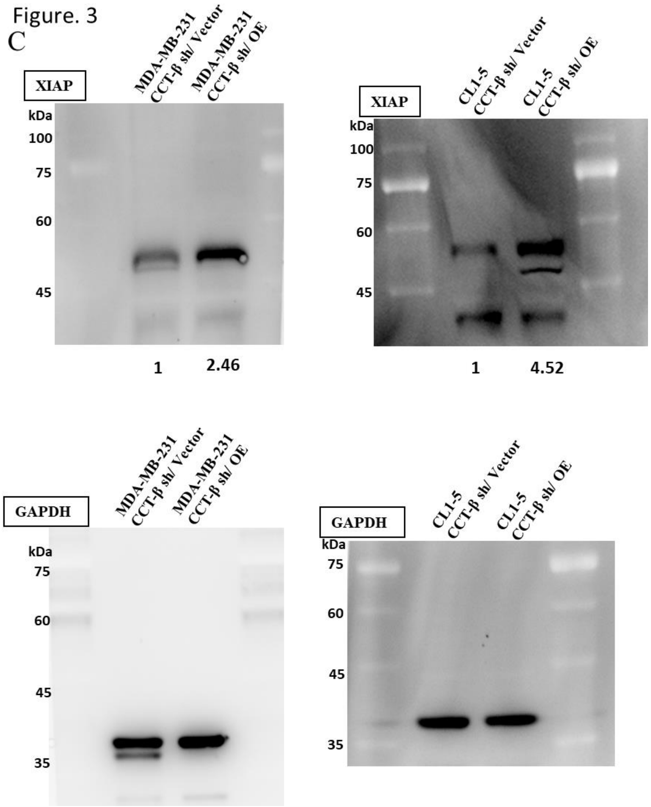

Figure. 3

D

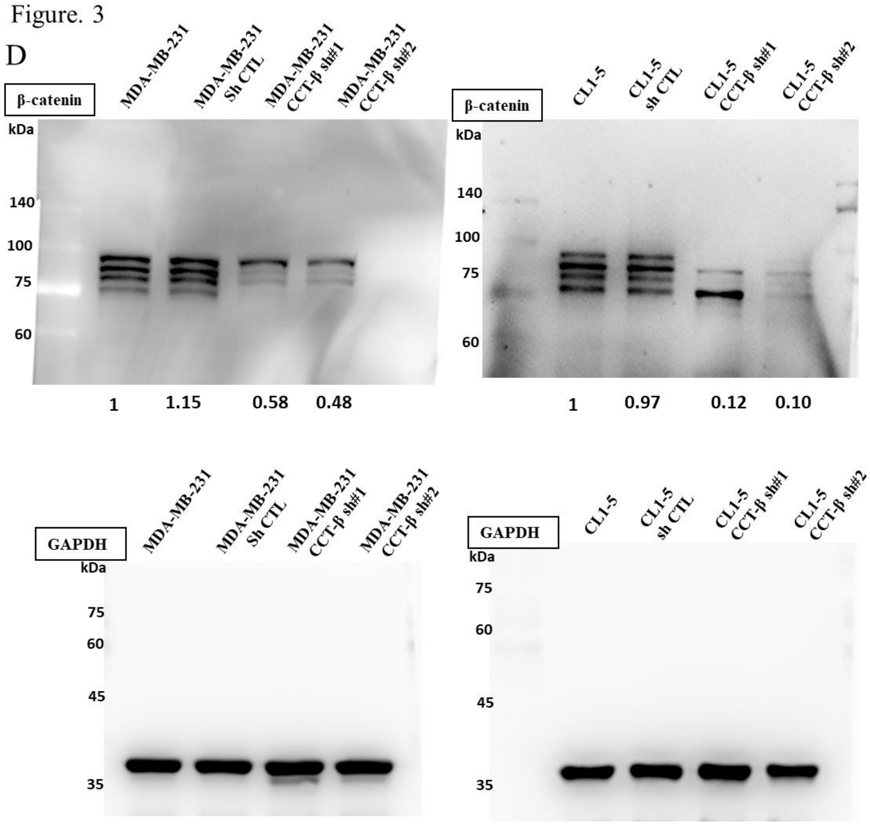

Figure. 3

E

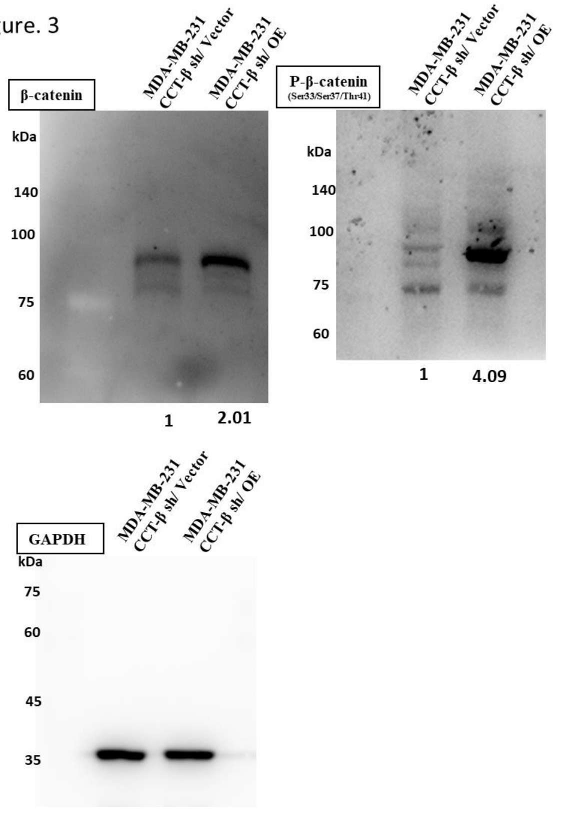

Figure. 3

E

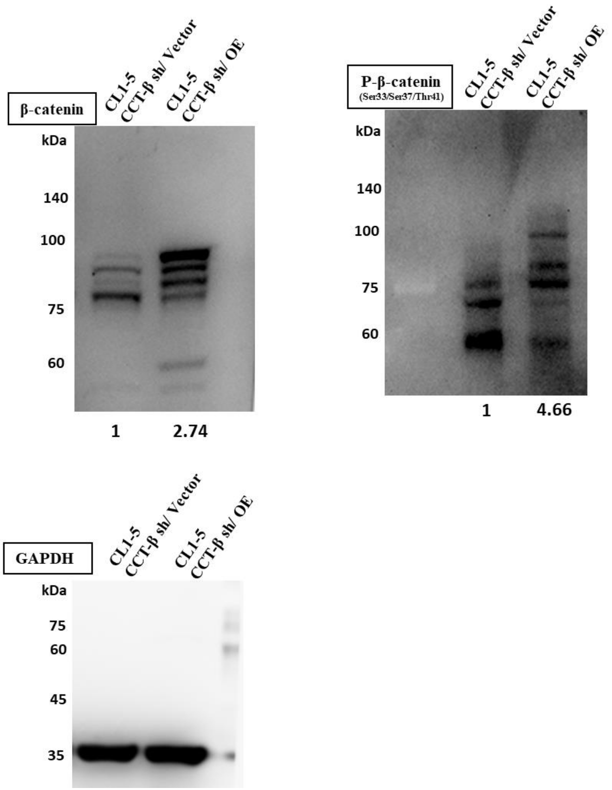

Figure. 4

A

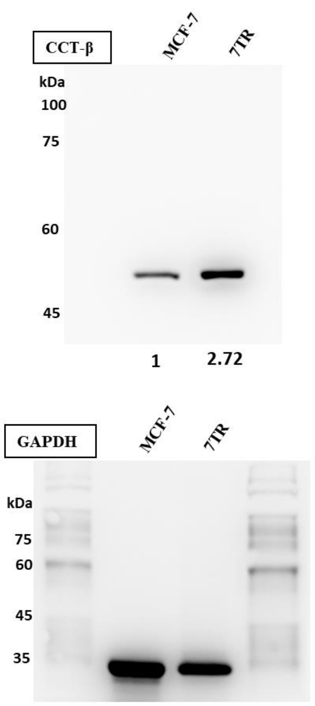

Figure. 4

C

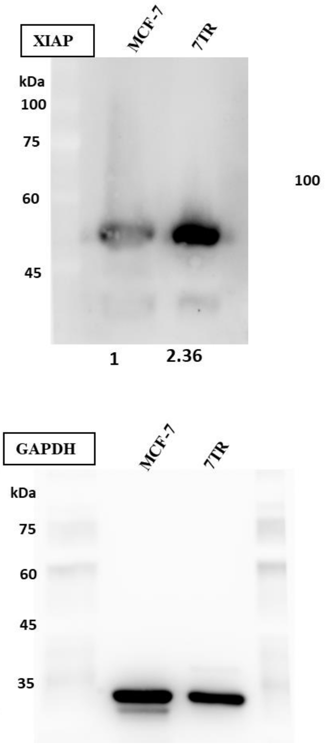

Figure. 4

C

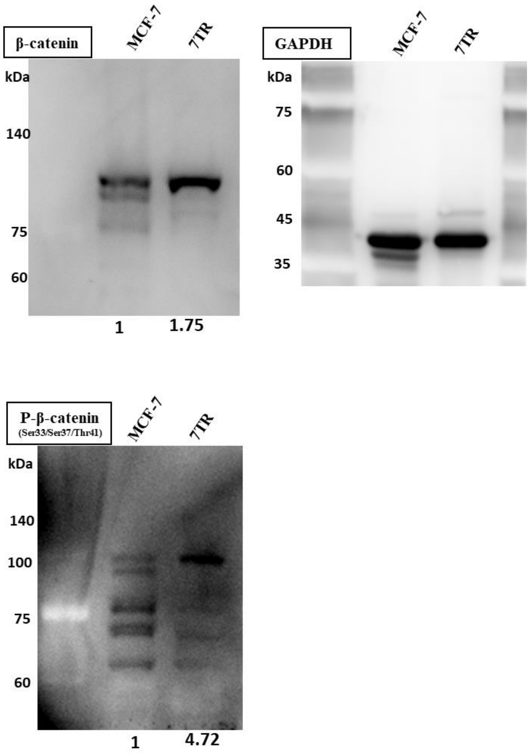

Figure. 4

D

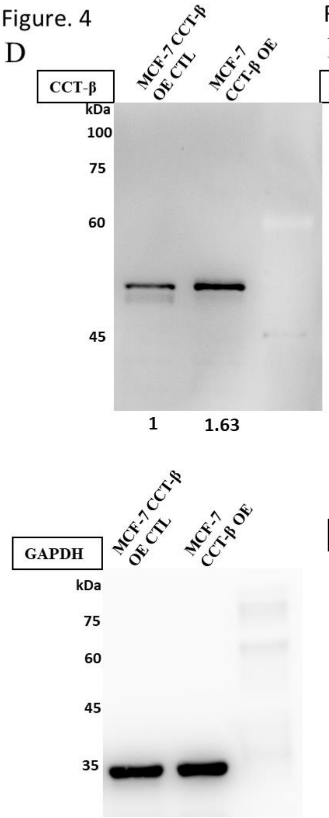

Figure. 4

F

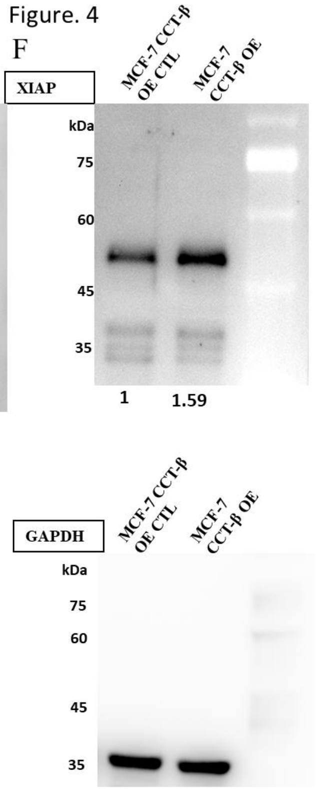

Figure. 4

F

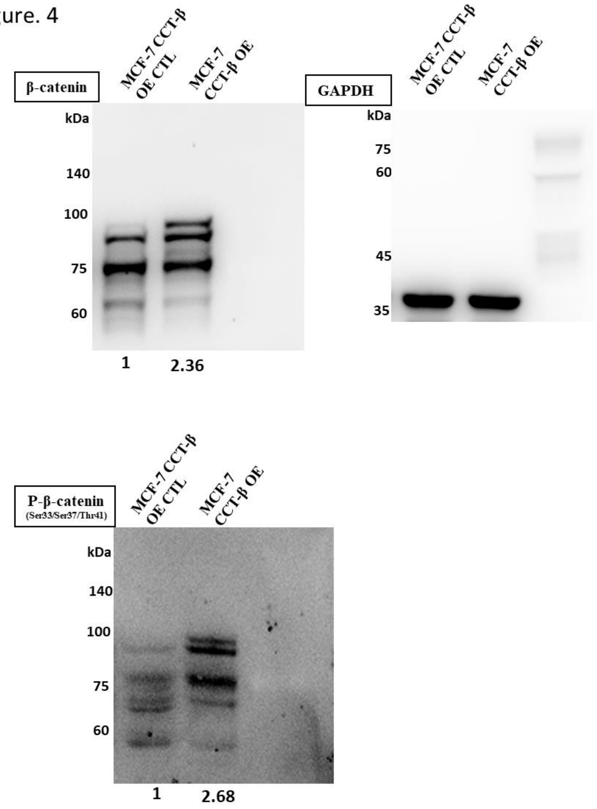

Figure. 4

G

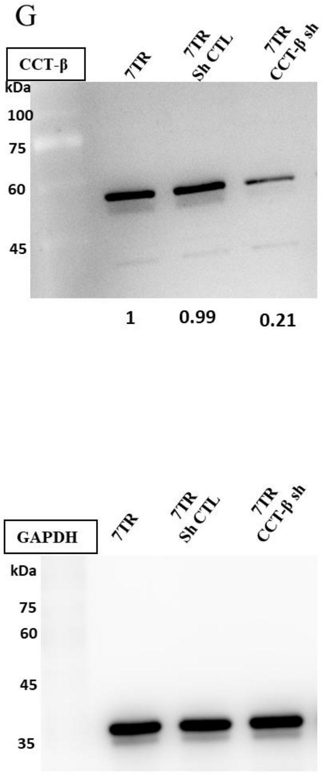

Figure. 4

I

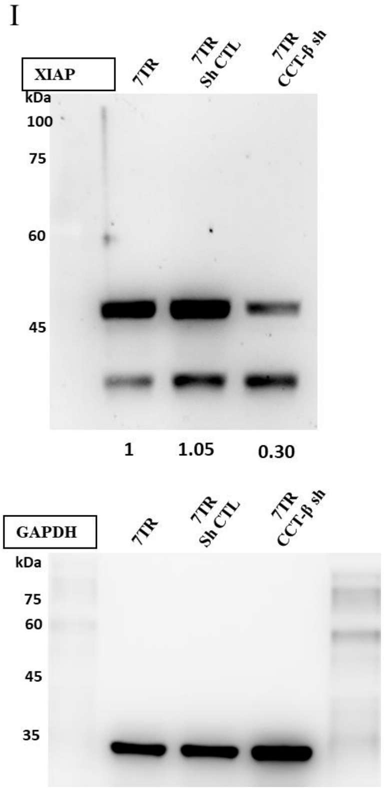

Figure. 4

I

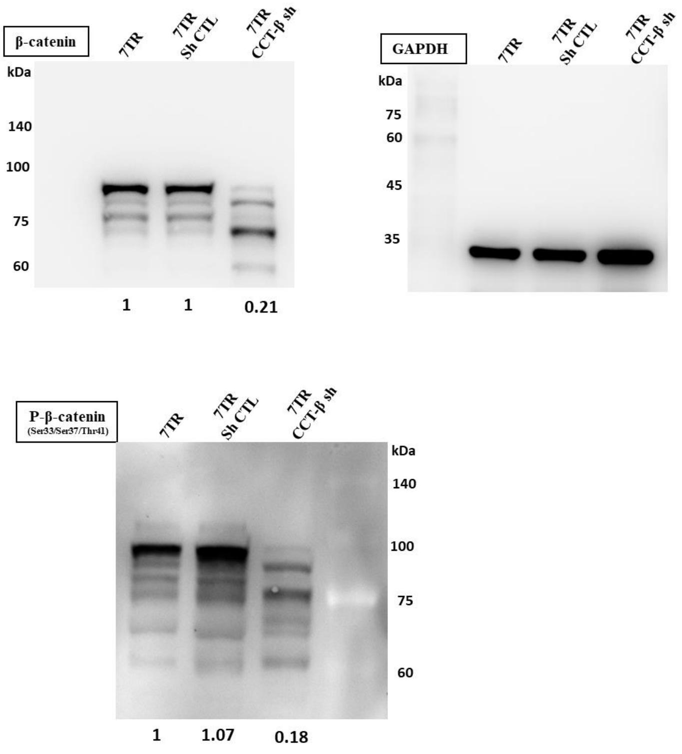

Figure. 7

B

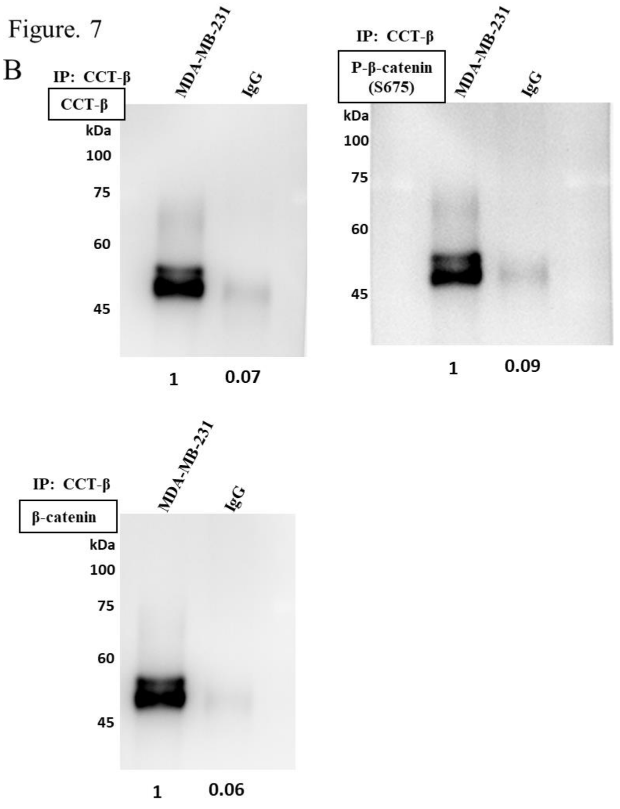

Figure. 7

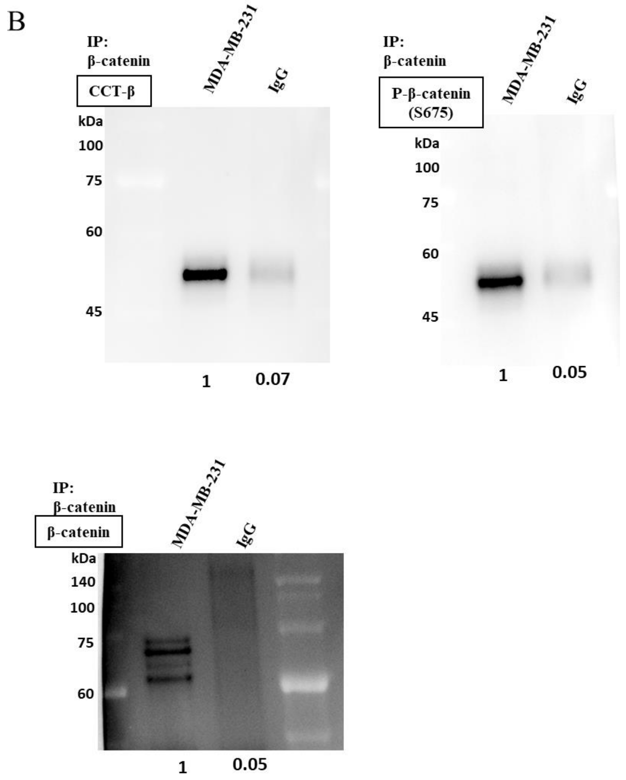

Figure. 7

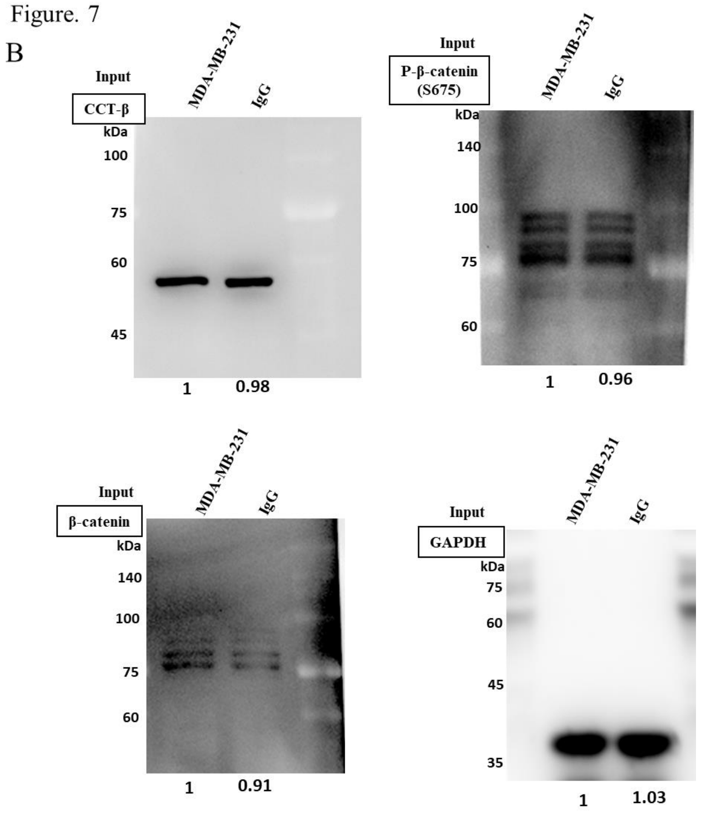

Figure. 7

B

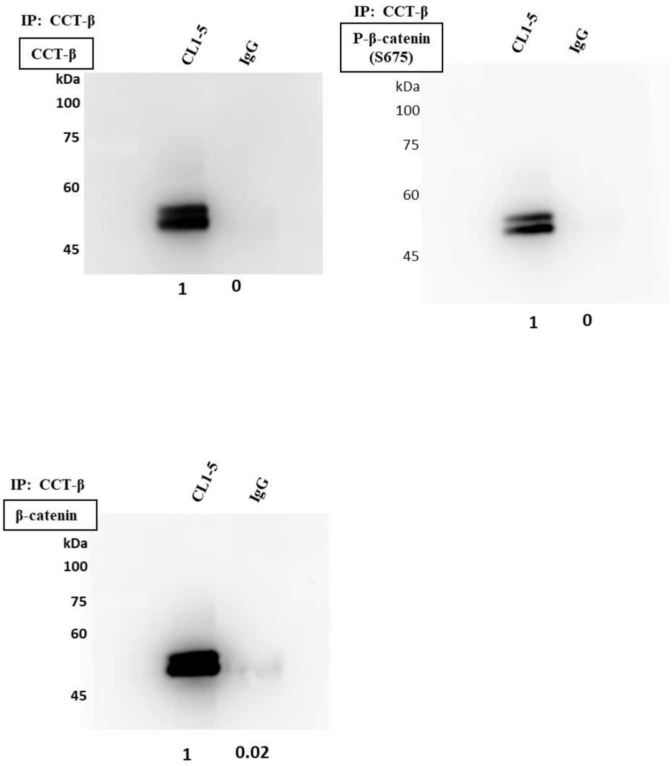

Figure. 7

B

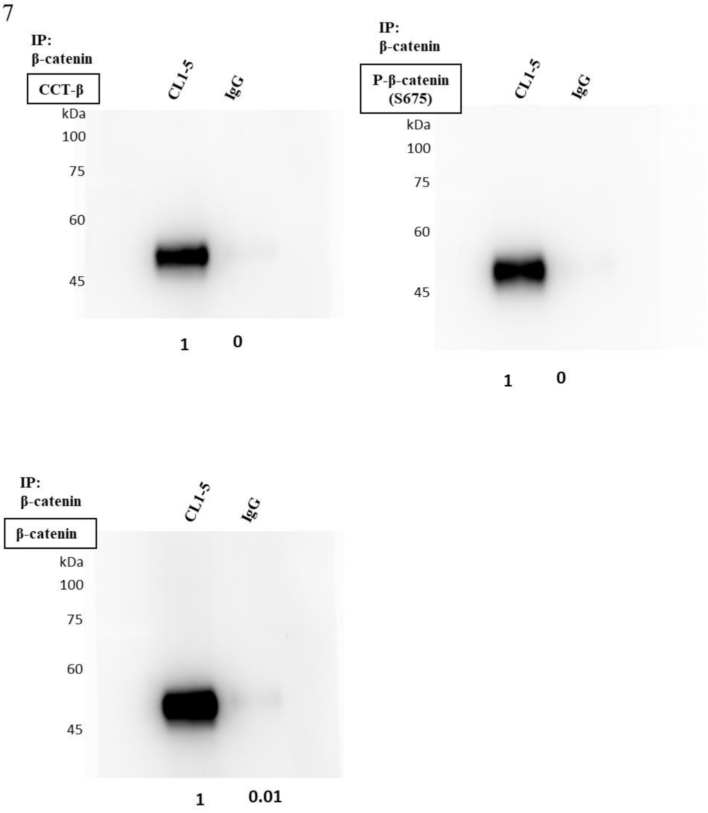

Figure. 7

B

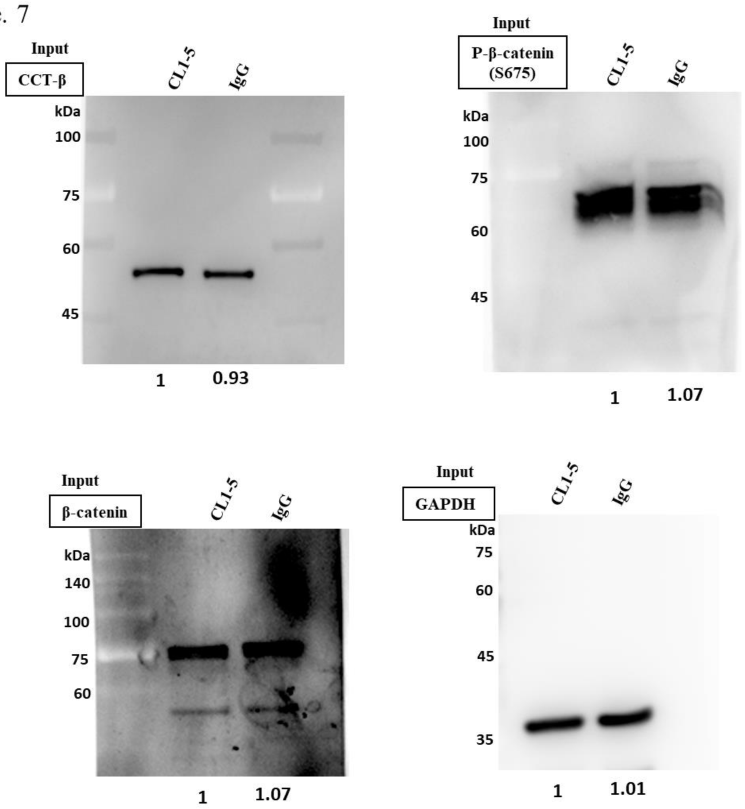

Figure. 7

C

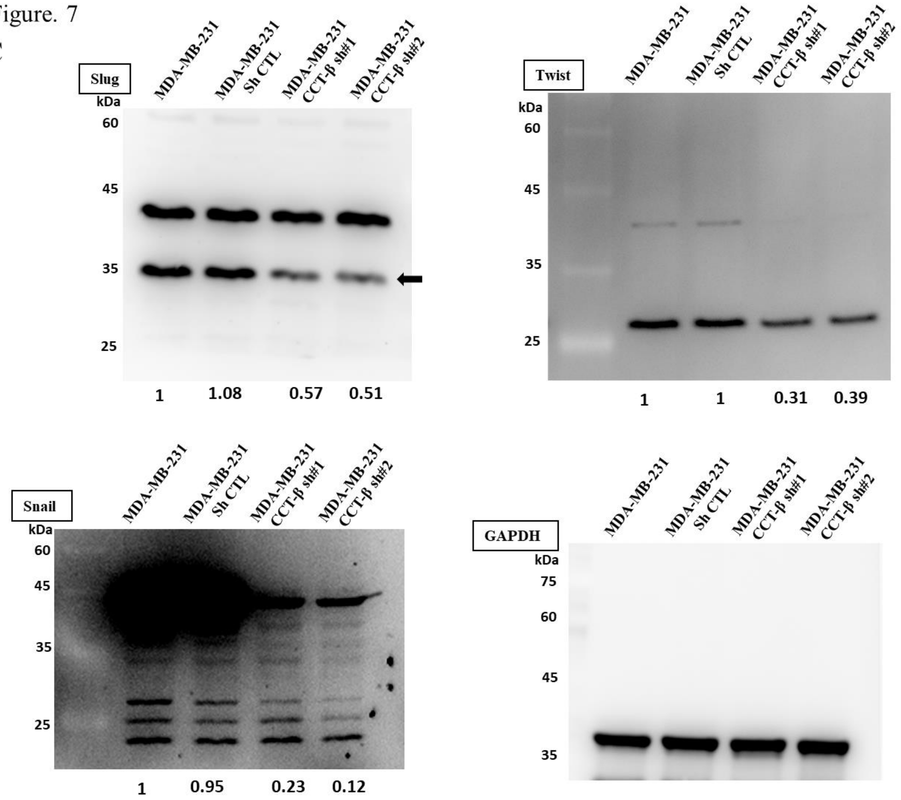

Figure. 7

C

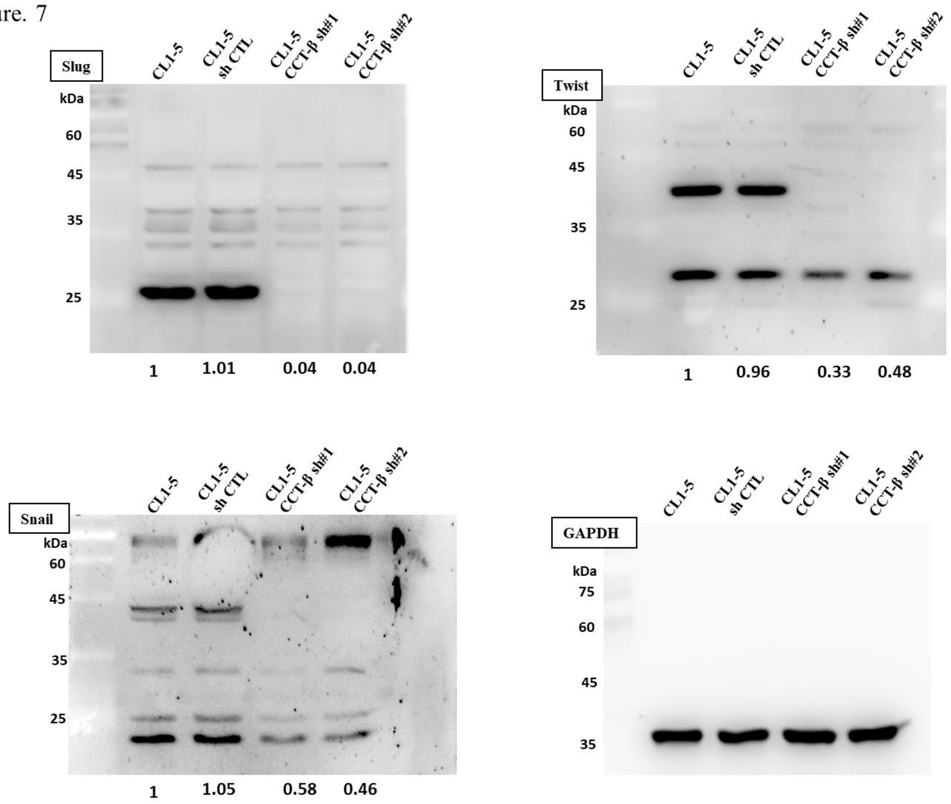

Figure. 7

D

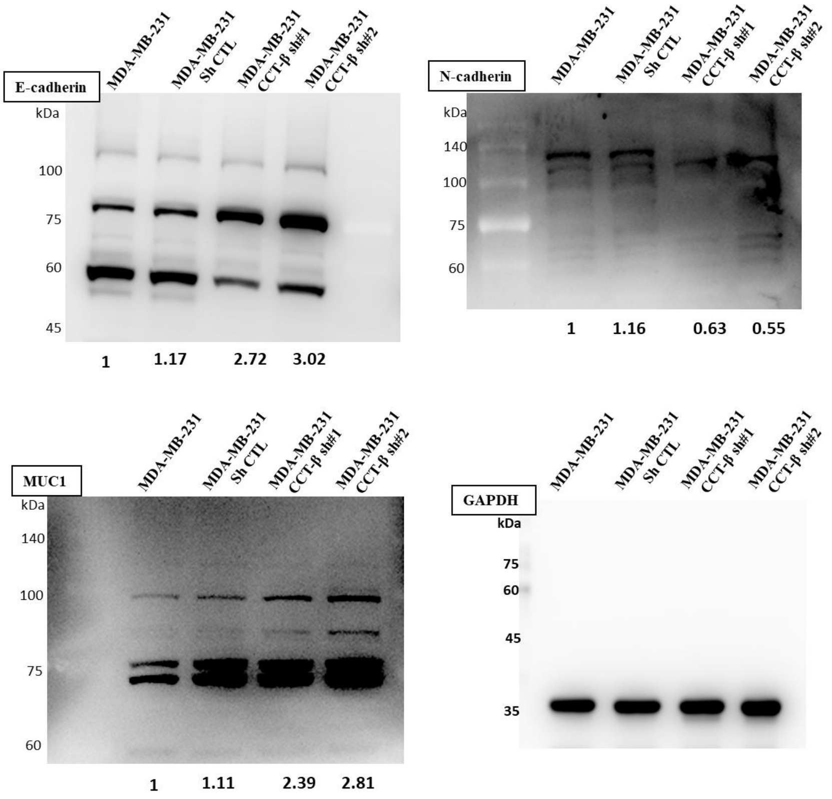

Figure. 7

D

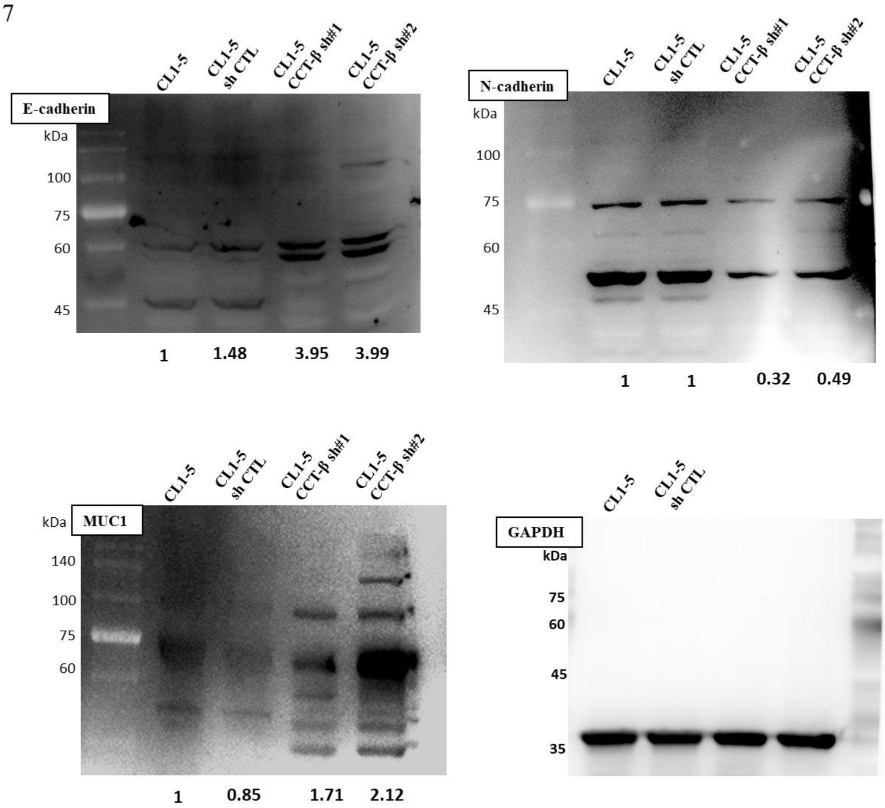

Figure. 7

E

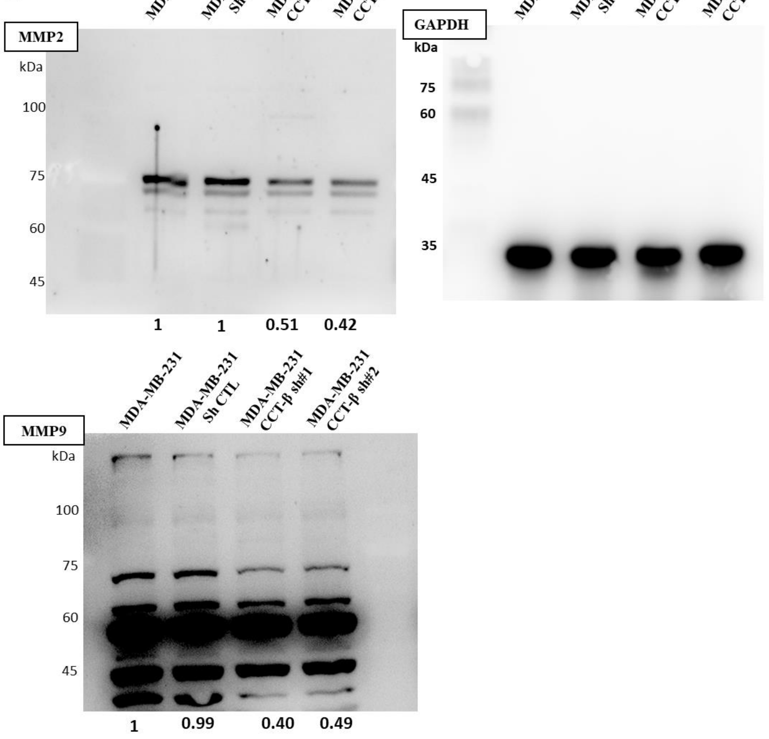

Figure. 7

E

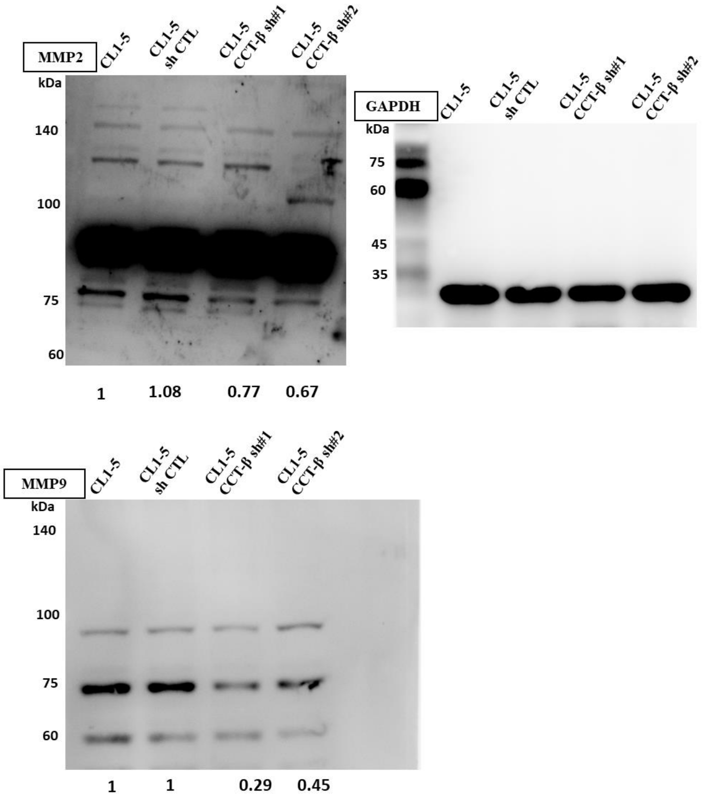

Figure. 7

F

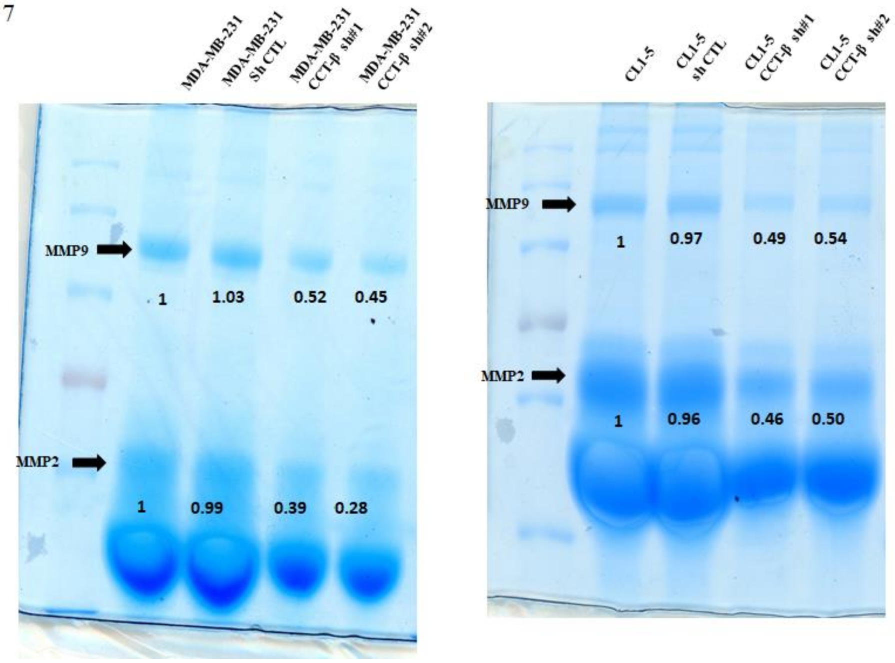

Figure. 8

A

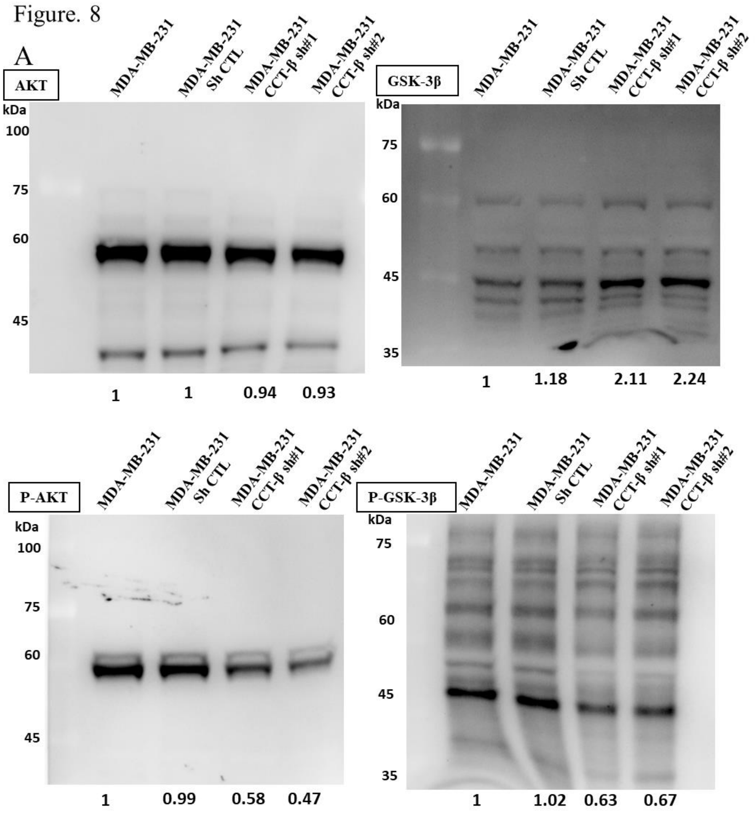

Figure. 8

A

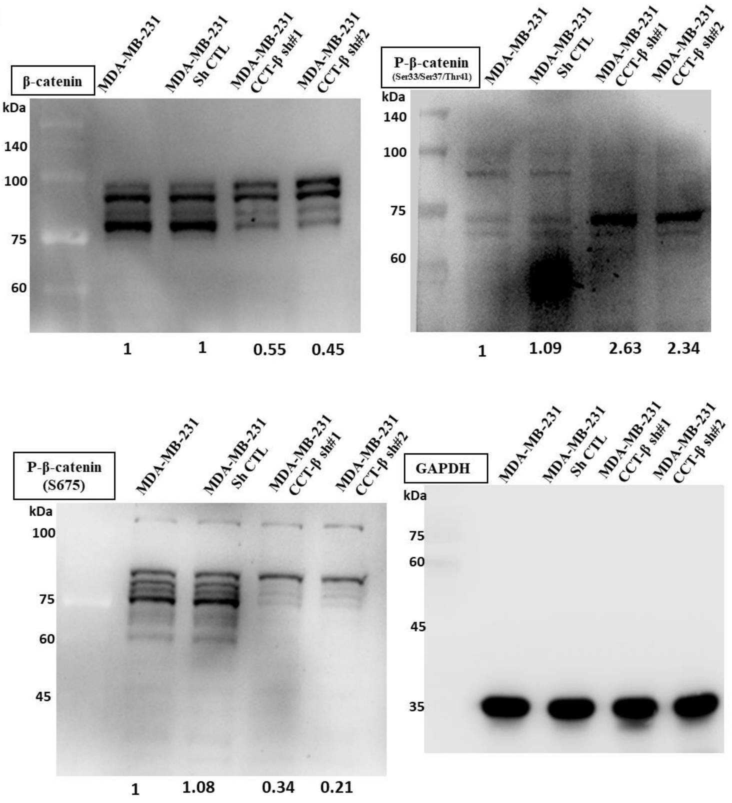

Figure. 8

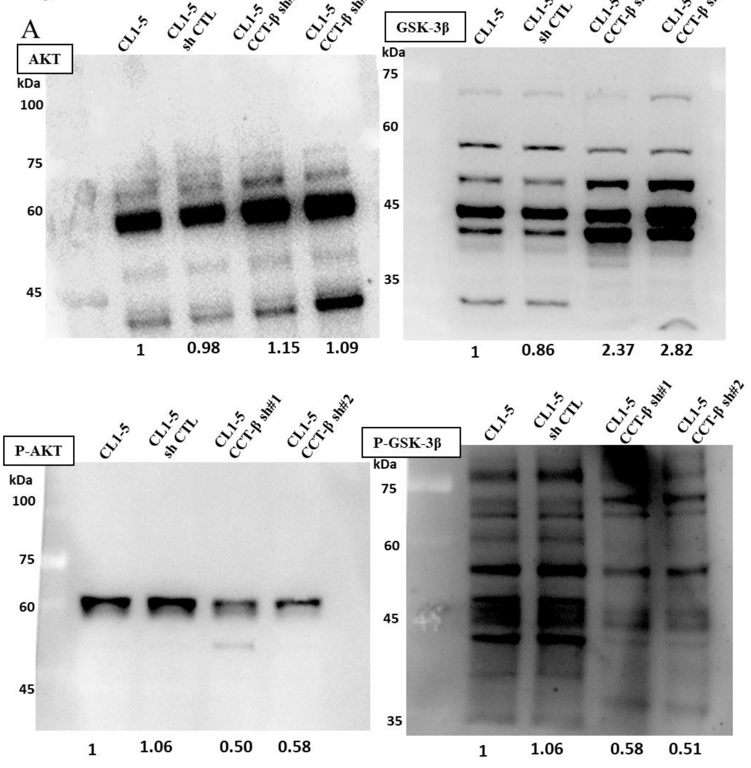

Figure. 8

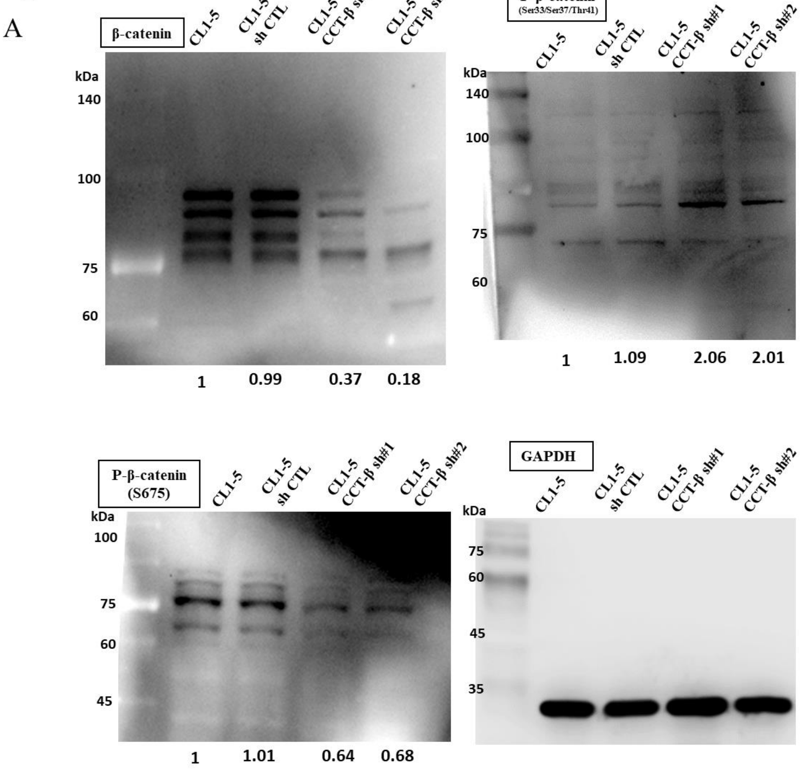

Figure. 8

B

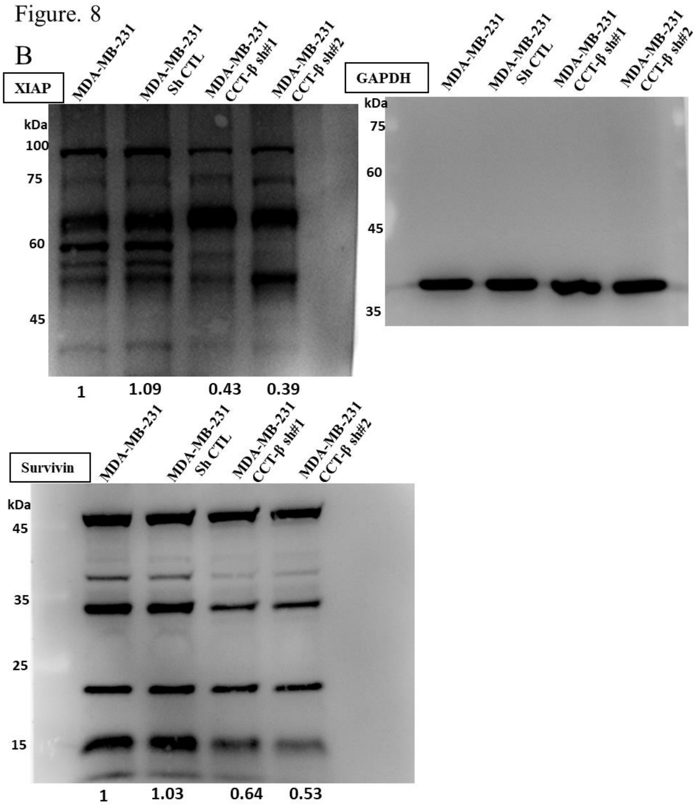

Figure. 8

B

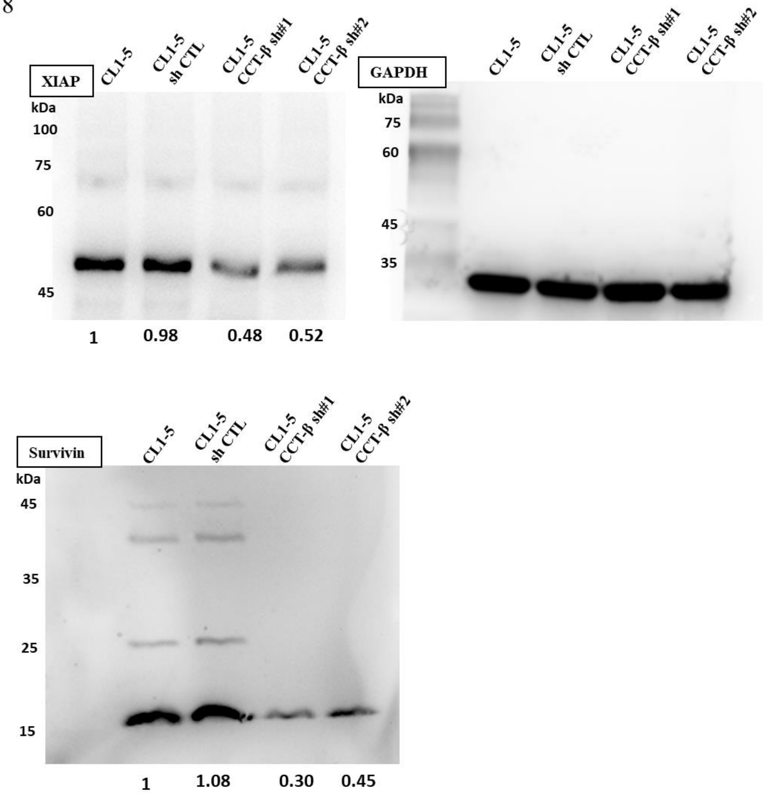

Figure. 8

C

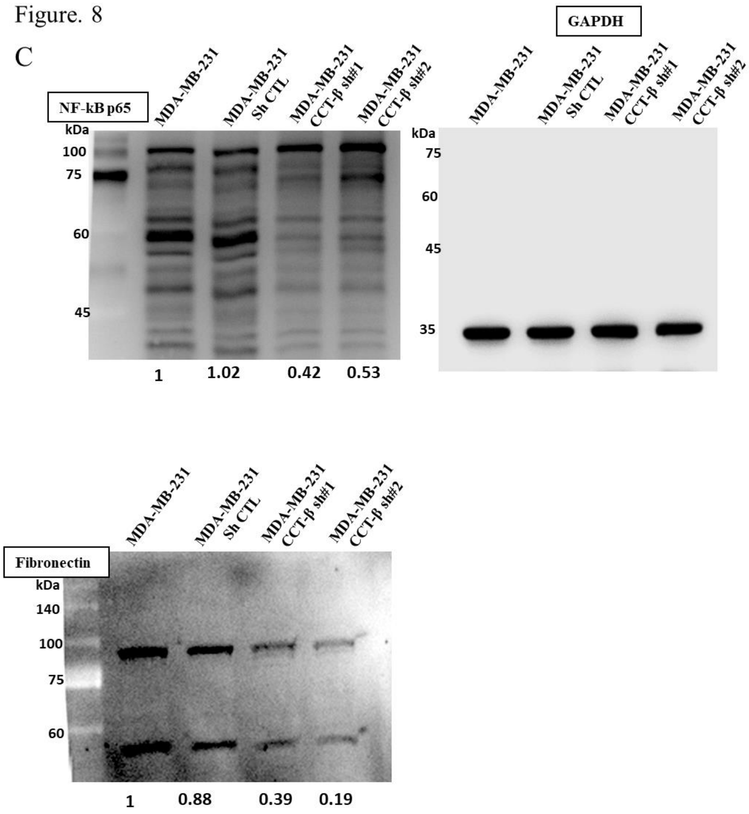

Figure. 8

C

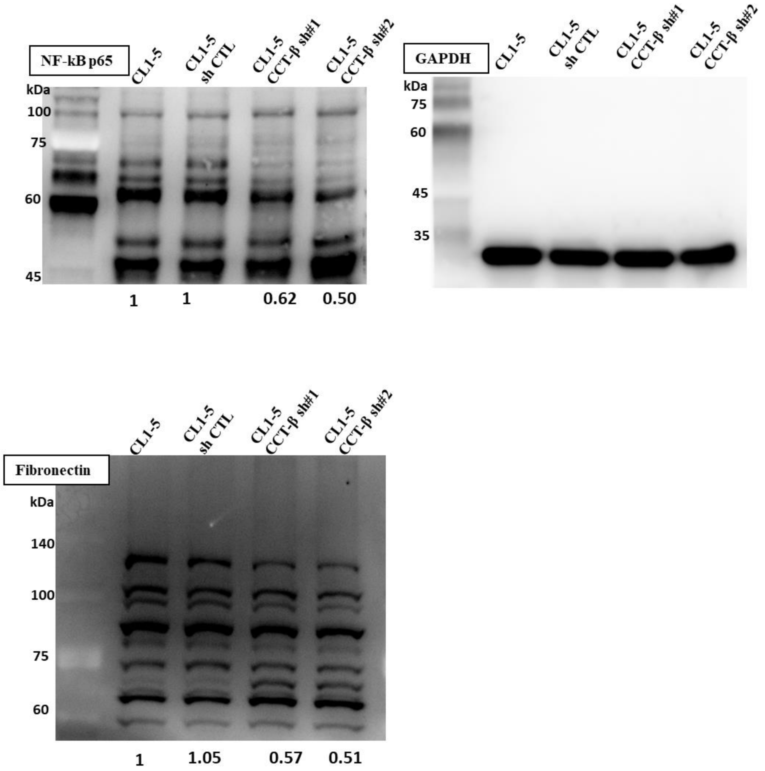

Figure. 8

D

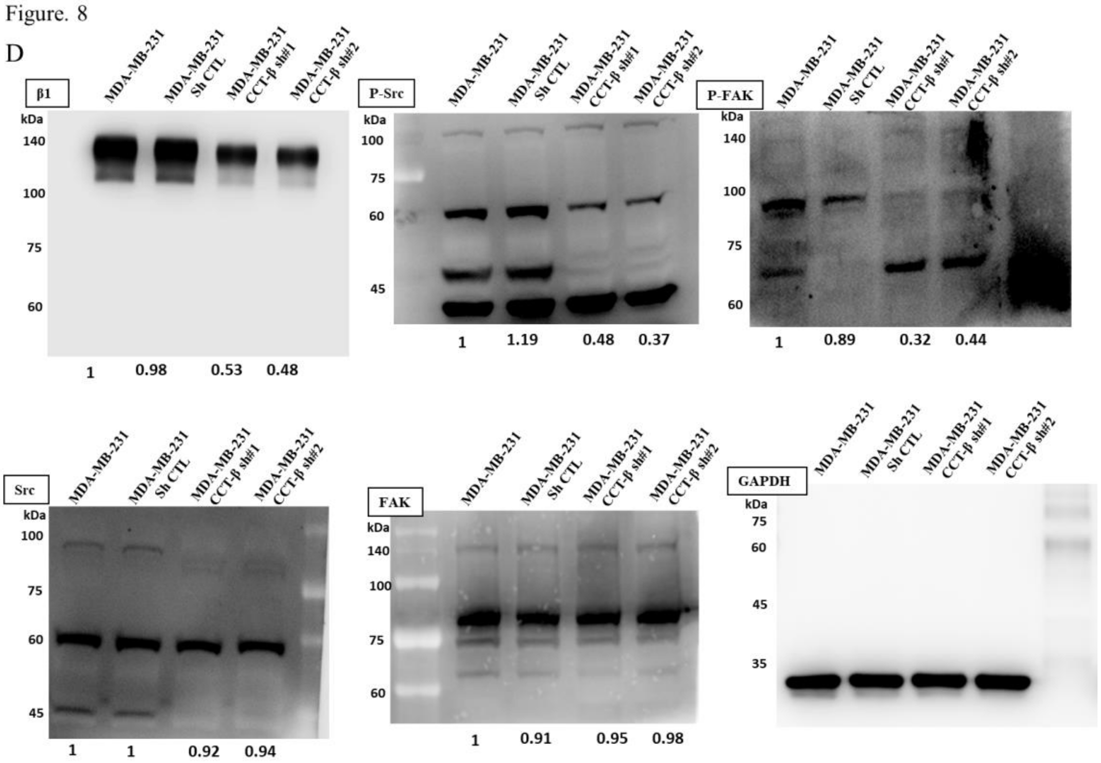

Figure. 8

D

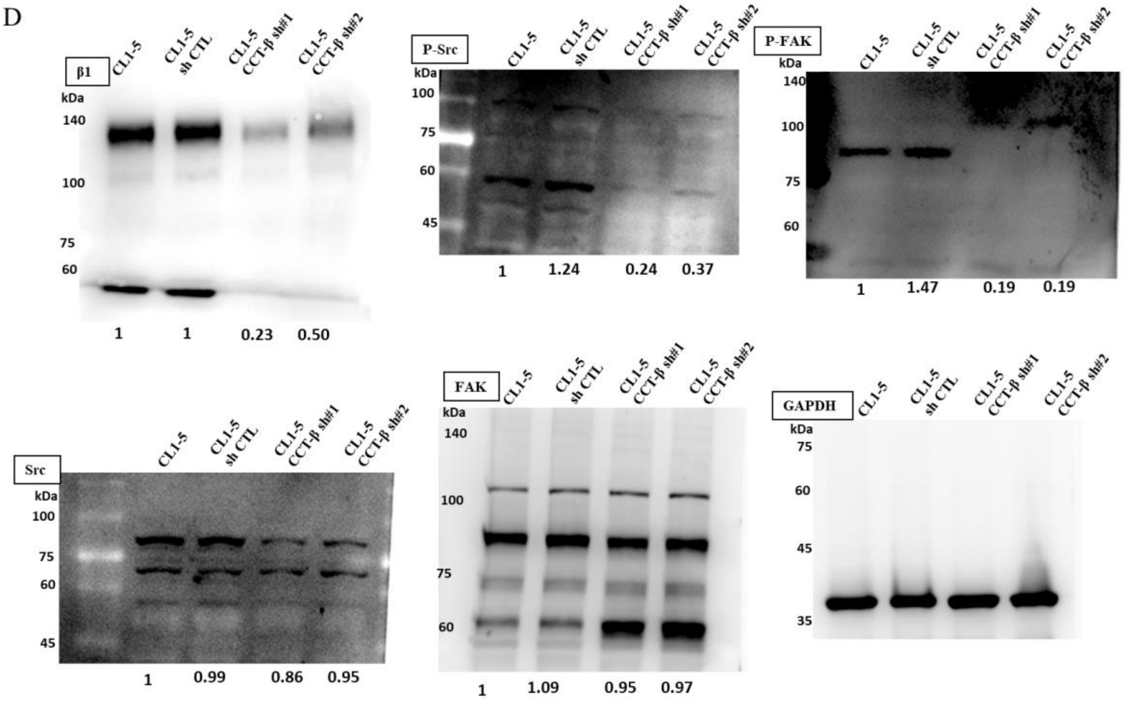

Figure. 8

E

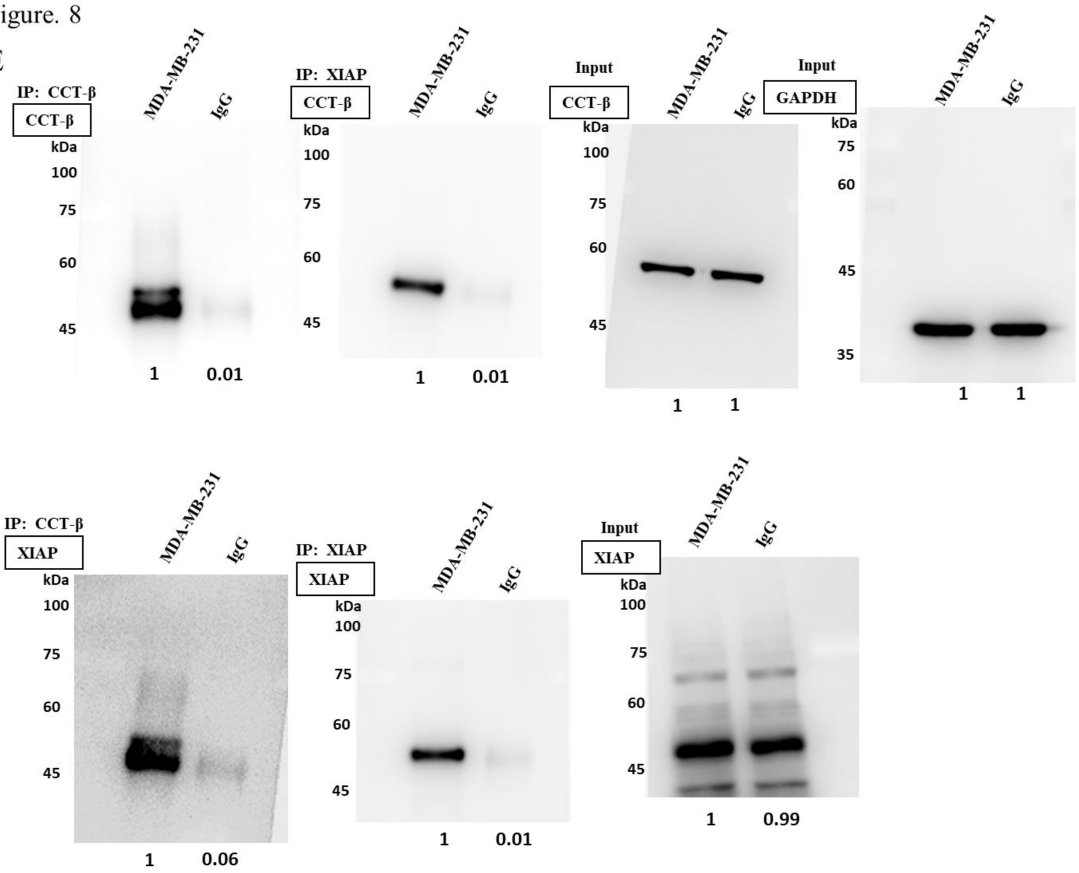

Figure. 8

E

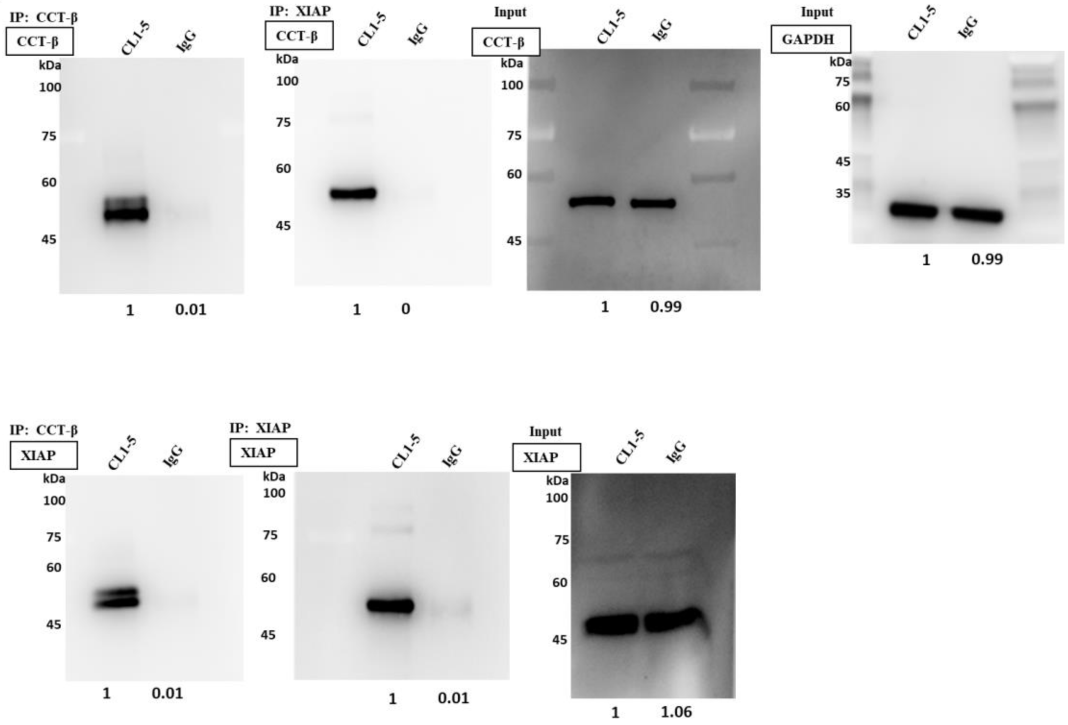

Figure S8. Uncropped western blots. Original western blots of Figs. 2, 3, 4, 7, and 8 with intensity ratios of the bands quantitated by densitometry readings**.** Quantitative analyses of the proteins presented were carried out using ImageJ software and were normalized with the loading control glyceraldehyde-3-phosphate dehydrogenase (GAPDH).
